# Peptide aromatic interactions modulated by fluorinated residues: Synthesis, structure and biological activity of Somatostatin analogs containing 3-(3′,5′difluorophenyl)-alanine

**DOI:** 10.1038/srep27285

**Published:** 2016-06-07

**Authors:** Pablo Martín-Gago, Álvaro Rol, Toni Todorovski, Eric Aragón, Pau Martin-Malpartida, Xavier Verdaguer, Mariona Vallès Miret, Jimena Fernández-Carneado, Berta Ponsati, Maria J. Macias, Antoni Riera

**Affiliations:** 1 Institute for Research in Biomedicine (IRB Barcelona). The Barcelona Institute of Science and Technology, Baldiri Reixac, 10, Barcelona 08028, Spain; 2 Departament de Química Orgànica, Universitat de Barcelona, Martí i Franqués, 1-11, Barcelona 08028, Spain; 3 BCN Peptides S.A. Pol. Ind. Els Vinyets-Els Fogars, Sector II. Ctra. Comarcal 244, Km. 22, 08777 Sant Quintí de Mediona, Barcelona 08777, Spain; 4 Institució Catalana de Recerca i Estudis Avançats (ICREA), Passeig Lluís Companys, 23, Barcelona 08010, Spain

## Abstract

Somatostatin is a 14-residue peptide hormone that regulates the endocrine system by binding to five G-protein-coupled receptors (SSTR1–5). We have designed six new Somatostatin analogs with L-3-(3′,5′-difluorophenyl)-alanine (Dfp) as a substitute of Phe and studied the effect of an electron-poor aromatic ring in the network of aromatic interactions present in Somatostatin. Replacement of each of the Phe residues (positions 6, 7 and 11) by Dfp and use of a D-Trp8 yielded peptides whose main conformations could be characterized in aqueous solution by NMR. Receptor binding studies revealed that the analog with Dfp at position 7 displayed a remarkable affinity to SSTR2 and SSTR3. Analogs with Dfp at positions 6 or 11 displayed a π-π interaction with the Phe present at 11 or 6, respectively. Interestingly, these analogs, particularly [D-Trp8,L-Dfp11]-SRIF, showed high selectivity towards SSTR2, with a higher value than that of Octreotide and a similar one to that of native Somatostatin.

Somatostatin (somatotropin release-inhibiting factor or SRIF) is a natural peptide hormone involved in multiple biological functions^1^. Although it has a pharmacological application as an anti-secretory drug, its use in growth hormone secretion disorders and in endocrine tumor treatment is hampered by its short half-life (2–3 min in plasma)^2^,^3^,^4^,^5^. The biological activity of Somatostatin is mediated by its high binding affinity to five structurally related G-protein-coupled receptors, named SSTR1–5^6^,^7^. The pioneering work of Vale *et al*.^8^ established that residues 7 to 10 form the pharmacophore region of Somatostatin. This finding fostered the development of a myriad of short-ring analogs, Octreotide (Sandostatin^®^)^9^,^10^ being the most successful, although others such as Lanreotide (Somatuline^®^)^11^, Vapreotide (Sanvar^®^)^12^, and Pasireotide (Signifor^®^)^13^ have also reached the market. Structural studies of short-ring analogs have been performed using NMR and X-ray techniques^14^,^15^,^16^,^17^,^18^. In addition, early NMR studies on the 14-amino acid native hormone identified an aromatic interaction between Phe6 and Phe11 (Fig. 1)^19^; however, the structure of the hormone could not be determined in solution due to the presence of several conformations in equilibrium^20^,^21^. In a project devoted to finding new Somatostatin analogs with improved stability and selectivity, we found that the replacement of one of the Phe residues in the 14-residue sequence by Msa (3-mesitylalanine) resulted in highly selective compounds^22^. Moreover, some of these new 14-residue peptides showed high conformational stability (particularly those that also had a D-Trp in position 8), thereby allowing the determination of their 3D structures by NMR for the first time, in a similar way as previous studies devoted to short-ring analogs, including Octreotide. These structures confirmed the relevance of non-covalent interactions between aromatic residues in positions 6, 7 and 11 in the modulation of the conformation and binding behavior of the correspondent Somatostatin analogs^22^,^23^,^24^.

Site-directed introduction of fluorinated amino acids has become an increasingly attractive approach in protein and peptide engineering^25^. Fluorinated amino acids cause minimal perturbation of the parent structures, but favor protein folding and stability due to their increased aromatic stacking and hydrophobicity^26^. However, to the best of our knowledge, the effect of introducing fluorinated Phe residues in the context of Somatostatin has not been addressed to date^27^. One of the few reports of Somatostatin analogs containing electron-poor aromatic residues as Phe isosteres was done by Hirschmann and co-workers^28^, who synthesized analogs containing L-pyrazinylalanine (Pyz).

In order to explore the role of aromatic interactions in the structure and function of Somatostatin analogs and to design new selective and stable compounds, we substituted the Phe amino acids of the natural sequence for fluorinated derivatives. The logic of this substitution involves the application of electron-poor aromatic side-chains (such as fluorinated phenylalanine), thus favoring the association with the electron-rich ring of native Phe residues. To test this hypothesis, we selected the L-3-(3′,5′-difluorophenyl)alanine (Dfp) from the fluorinated amino acids available. Since we aimed to characterize these interactions using NMR, the Dfp analog has the advantage that its aromatic ring still holds three hydrogen atoms—a valuable feature that provides structural information via NOE interactions with neighboring residues. Here we describe the synthesis and NMR structures of six new Somatostatin analogs containing Dfp amino acid. We also analyze the binding preferences of these new analogs with respect to SSTR1–5.

## Results

### Design of six new Somatostatin analogs (peptides 1–6)

**Figure 1:**
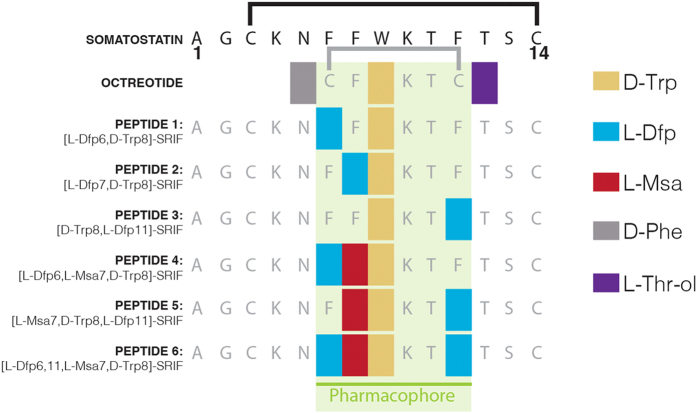
Sequences of Somatostatin and Octreotide and of the six Somatostatin analogs synthesized in this work. For simplicity, the disulfide bridge present in these molecules is displayed only on the top of the Somatostatin and Octreotide sequences. The modified amino acids are depicted as colored rectangles, yellow for D-Trp, blue for L-Dfp, red for L-Msa, gray for D-Phe, and purple for L-Thr-ol. The pharmacophore region of the native hormone and of all analogs is highlighted in green.

Most of the Somatostatin analogs developed to date are short-ring cyclic peptides that contain the pharmacophore region comprising the residues —FFWKTF— of the hormone. In contrast, our approach was to introduce small modifications into its natural 14-amino acid sequence, since the remaining amino acids also play a role in the biological activity of the hormone^22^. All our peptides were designed to contain a D-Trp because the replacement of L-Trp in position 8 by its D enantiomer enhances the stability of the analogs while maintaining similar binding affinities^2^,^29^,^30^,^31^,^32^. The sequences of the six analogs studied herein are shown in Fig. 1. In the first group, we replaced, one by one, Phe6, Phe7 and Phe11, respectively, by Dfp (peptides **1–3**). We previously observed that replacing Phe7 by Msa favors the aromatic interaction between the benzene rings of Phe6 and Phe11^22^. Therefore, we designed two more analogs that combined Msa in position 7 and Dfp in positions 6 or 11 (peptides **4** and **5**). Finally, an analog with both Phe6 and Phe11 replaced by Dfp (peptide **6)** was also synthesized in order to test the effect of two fluorinated residues in the presence of Msa7.

### Synthesis of Fmoc-L-Difluorophenylalanine (Fmoc-L-Dfp-OH) and the six peptides

**Figure 2:**
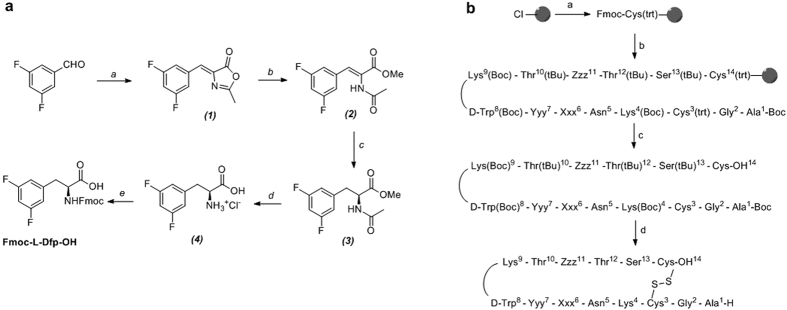
Schematic representations of the Dfp synthesis and the synthetic protocol used for the preparation of peptides **1–6**. (**a**) Synthesis of Fmoc-L-Dfp-OH. *a*) *N*-Ac-Gly-OH, AcONa, Ac_2_O, 2 h, 100 °C; *b*) MeONa, MeOH, 2 h, 70 °C; *c*) [Rh(*S*-MaxPHOS)(COD)]BF_4_ cat 3%, H_2_ (5 bar), MeOH, r.t.; *d*) HCl aq, 6 h, reflux; *e*) FmocOSu, Na_2_CO_3_, H_2_O, acetone, 0 °C to r.t.; (**b**) General schematic synthesis pathway for peptides. a) 1. Fmoc-L-Cys(trt)-OH (3 eq.), DIPEA (4 eq.), 2. MeOH; b) 1. Piperidine 20% DMF, 2. Fmoc-Aaa-OH (1.5–3 eq.), DIPCDI (3 eq.), HOBt (3 eq.), DMF, c) CH_2_Cl_2_/TFE/AcOH, d) 1. I_2_, 2. TFA/CH_2_Cl_2_/anisole/H_2_O.

Dfp was synthesized starting from 3,5-difluorobenzaldehyde, as described in Fig. 2a. The azlactone (***1***) was obtained by treatment with *N*-acetylglycine and the subsequent ring-opening reaction with (MeONa/MeOH) produced the (*Z*)-methyl-2-acetamido-3-(3′,5′-difluorophenyl)acrylate (***2***) in 58% overall yield (>10 g scale). The (*E*)-isomer was not detected by TLC or by ^1^H-NMR. Using an asymmetric hydrogenation reaction with [Rh(*S*-MaxPHOS)(cod)]BF_4_^33^,^34^ as hydrogenation catalyst, we prepared the acetamido ester (***3***) with 99% ee (*S*)-enantiomer. Fmoc-L-Dfp-OH was obtained in excellent yield and optical purity after acid hydrolysis of (***3***) (to give ***4***) and final Fmoc-protection^22^. Peptides **1–6** (Fig. 1) were prepared by standard Fmoc/*t*Bu solid-phase peptide synthesis (SPPS)^35^ using 2-chlorotrityl chloride resin^22^,^23^,^24^. The schematic representation of the synthetic strategy used for the six peptides is shown in Fig. 2b. TFA salts of the purified peptides were used in the NMR studies, but the TFA anions were exchanged for acetates prior to receptor binding studies.

### Affinities of the new Somatostatin analogs towards SSTR1–5

For each peptide **1–6**, the binding affinity towards SSTR1–5 was measured by a competitive binding assay using ^125^I-labeled Somatostatin-14 and various concentrations of each unlabeled ligand, respectively, in triplicate. For comparative purposes, the same test was applied to Somatostatin and Octreotide. The K_i_ values were calculated using the Cheng and-Prusoff equation (Table 1)^36^. The receptor-subtype selectivity profiles of these analogs will be discussed in detail in parallel with the details of the structural studies.

### NMR studies of Peptides 1–6

Lyophilized peptides were dissolved and neutralized in aqueous solution (1 mM concentration) and analyzed by NMR. In all cases, well-dispersed two-dimensional NOESY and TOCSY homonuclear experiments showed a major set of NOE peaks, whose assignments enabled us to characterize the main conformations of the peptides in solution. Structures were generated using the Crystallography & NMR System (CNS)^37^ protocols.

### [L-Dfp6,D-Trp8]-SRIF (peptide 1)

**Figure 3:**
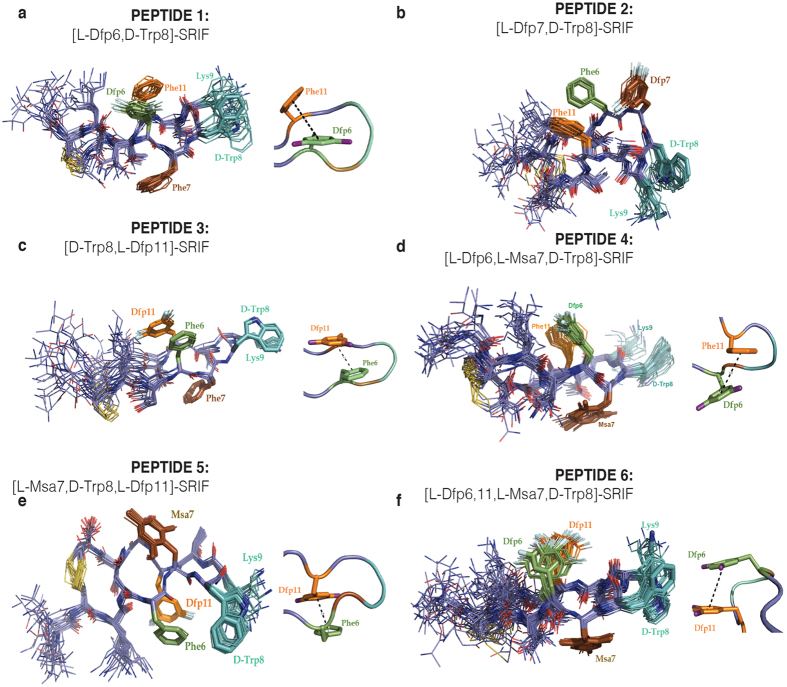
Superimposition of the 20 best conformers selected on the basis of the low energy values of the six Somatostatin analogs, namely peptide **1–6** (shown as panels **a** to **f** respectively). Next to these figures we show their π−π aromatic interaction between aromatic residues in position 6 and 11. Residues are highlighted by position.

The 2D NMR spectra showed a large number of NOE signals that have been unambiguously assigned. This information was used as restrictions to characterize the most populated conformations for this peptide in aqueous solution. These conformations are shown in Fig. 3a as a superimposition of a family of conformations ordered on the basis of their energy. The most remarkable features of this family of conformations were the hairpin involving residues 6–11 and the aromatic stacking between Dfp6 and Phe11. The hydrophobic interaction between D-Trp8 and Lys9, which further stabilizes the hairpin area, was defined by numerous proton-proton contacts between these two residues. In addition, most of the NOE contacts displayed by the Dfp6 side-chain were with the Phe11 side chain (Hδ of Phe11 with the Hα, Hβ2, Hβ3 and Hδ of Dfp6 and Hε of Phe11 with Hβ2, Hβ3, Hδ and Hζ of Dfp6). The π−π interaction between Dfp6 and Phe11 was found to belong to the *offset-stacked* type, but the aromatic rings were not oriented parallel to one another. As previously described in the models reported by Hunter and Sanders^38^ and by Cozzi, Siegel *et al*.^39^, the poor π-electron density of the difluorophenyl ring reduced the electrostatic repulsion between the π-electron clouds of Dfp6 and Phe11, thereby enhancing the π-stacking interaction between them. In peptide **1**, the Phe7, placed at the opposite side of the molecule, did not participate in these aromatic interactions. Indeed, the aromatic ring of Phe7 has contacts with the Hβ2 of Asn5 and with the Qγ2 methyl groups of Thr10 and Thr12. Consequently, the conformations of peptide **1** are more restricted than the counterpart without the fluorine substitution (D-Trp8-SRIF) previously analyzed^22^. The binding profile of peptide **1** showed strong affinity to SSTR2, similar to that of Octreotide, and remarkable affinity to SSTR3, in the same order as Somatostatin.

### [L-Dfp7,D-Trp8]-SRIF (peptide 2)

The NMR data obtained for this peptide also showed remarkable conformational stability, thereby allowing the determination of its 3D structure in solution (Fig. 3b). In striking contrast to the previous structures, the lower energy conformations of peptide **2** showed all aromatic residues located on the same side of the molecule. The Dfp7 residue showed several NOE contacts with the Phe6 and Phe11 side-chains and result in an *edge-to-face* orientation of the aromatic rings. These NOEs were unambiguously assigned and involved the Hδ of Dfp7 and the Hα and Hβ2/β3 of Phe6 and also between the aromatic ring of Phe11 with the beta protons of Phe6 and Dfp7. The NMR data indicated that the aromatic side-chain of Dfp7 occupies a position near to the D-Trp8 residue. In order to increase the aromatic interactions with the fluorinated residue in 7, residues 6 and 11, moved from the top to the bottom of the molecule (according to the orientation shown in the Fig. 3b), probably due to the stronger π−π stacking tendency of the difluorobenzene ring. The indole ring displayed numerous NOE signals with the aliphatic side-chain of Lys9 as a result of the stabilizing effect of the D-Trp8-Lys9 hydrophobic interaction. Somewhat surprisingly, although the overall geometry of peptides **1** and **2** differed greatly, these molecules showed a similar binding profile. The affinity of these two peptides for SSTR2 was similar to that of the Octreotide used for comparison purposes.

### [D-Trp8,L-Dfp11]-SRIF (peptide 3)

The NMR data corresponding to this analog also displayed an abundant number of NOEs, thereby allowing us to calculate a family of well-defined 3D structures (Fig. 3c). NOEs between Phe6 and Dfp11 were both abundant and intense, indicating close proximity of their side-chains in solution. The aromatic moieties of these residues were engaged in a parallel *offset-stacked* aromatic interaction in which the most negatively charged part of the Dfp11 ring pointed away from the π-cloud of the native Phe6. This ring orientation, defined by several NOE contacts between Dfp11 and the side-chains of Lys4, Phe6, Thr12 and Ser13, was similar to that of the aromatic interaction between Dfp6-Phe11 in peptide **1**. In these cases, the Phe7 aromatic side-chain was accommodated on the other side of the molecule, thus facilitating the aromatic interaction between Phe6 and Dfp11. This analog displayed high affinity and selectivity towards SSTR2, presenting *K*_i_ values similar to Somatostatin, and improving that of Octreotide (Table 1).

### [L-Dfp6,L-Msa7,D-Trp8]-SRIF (peptide 4)

In previous studies^22^, we reported that the presence of a mesityl residue in position 7 enhances the π−π interaction between aromatic residues 6 and 11, thus providing more conformationally restricted peptides with high SSTR2 affinity. With the aim of reinforcing the Dfp6-Phe11 interaction, we designed a new analog including the Msa substitution at position 7. However, peptide **4** showed greater conformational mobility than expected, as revealed by NMR data. The π−π-aromatic interaction between Dfp6 and Phe11 was present with the offset-stacked displacement of the rings more pronounced than in the absence of Msa7 (Fig. 3d). The angle between rings was closer to an *offset-stacked* parallel geometry. As we have observed in other peptides, the Msa7 residue did not participate in the aromatic interactions with residues 6 and 11, since it was placed at the opposite side of the molecule. However, in peptide **4**, the mesityl ring was oriented towards the center of the molecule, as the equivalent phenylalanine of peptide **1**. The affinity for SSTR3 dropped one order of magnitude compared to peptide **1**, whereas that towards SSTR2 increased. Overall, the geometry of peptide **4** was similar to that of Somatostatin analogs with Msa in position 7, previously described by our group^22^,^23^,^24^. The main difference was that the π−π interaction was now *offset-stacked* instead of *edge-to-face*. Analog **4** showed high selectivity toward SSTR2 (with a Ki value in the same range as that of Octreotide), although its binding affinity was not as high as in the [Msa7,D-Trp8]-SRIF analog reported previously^22^. Nevertheless, the presence of Msa7 improved the pharmacological profile of the molecule since it increased selectivity towards SSTR2 by decreasing affinity to SSTR4 and SSTR5.

### [L-Msa7,D-Trp8,L-Dfp11]-SRIF (peptide 5)

The NMR data corresponding to the substitution of Phe7 for its Msa7 counterpart in the peptide **3** scaffold showed high conformational rigidity by NMR, thus allowing the identification of a set of low energy conformers superimposed and displayed in Fig. 3e. These structures showed that Dfp11 and Phe6 adopt an aromatic *face-to-face* orientation defined by an abundant set of unambiguously identified NOEs. Interestingly, peptide **5** had residues 6 and 11 on the opposite side with respect to the orientation observed for peptide **3**. Indeed, the general structure of **5** differed greatly from those characterized for peptides **1** and **4.** The binding of peptide **5** to SSTR2, SSTR3 and SSTR5 decreased by one order of magnitude with respect to that of peptide **3.** This observation may be attributed to the aforementioned differences. However, peptide **5** was still relatively selective for SSTR2 (with a Ki of the same order of magnitude as Octreotide). As occurred with peptide **4**, the replacement of Phe7 by Msa decreased the binding capacity to SSTR4 and SSTR5 with respect to the results obtained with peptide **3**.

### [L-Dfp6,11,Msa7,D-Trp8]-SRIF (peptide 6)

We also examined the effect of replacing both Phe6 and Phe11 by Dfp residues, maintaining the Msa7 and D-Trp8 residues, on the binding specificities of the Somatostatin analogs. On the basis of the NMR data, and despite the number of substitutions introduced, this peptide folded with the characteristic orientation that maintains residues 6 and 11 on one face of the structure and the Msa7 on the other—similar to the postulated SSTR2—bioactive conformation of Somatostatin^22^. A family of conformers selected on the basis of lower energy values were superimposed and are shown in Fig. 3f. The interaction between the pair of Dfp6 residues, which are more hydrophobic and electron-poor aromatic rings than the Phe6-Phe11 phenylalanine pair, provided more stabilization than the interaction present in the parent compound. This observation is in agreement with the electrostatic^26^ and the polar/π^27^ model where electron-withdrawing substituents enhance the π-stacking interactions because they decrease the aryl π-electron density, thus minimizing repulsion between two electron-poor rings. However, receptor-subtype assays for this analog revealed strong selectivity towards SSTR2 (probably induced by the presence of Msa7, which by itself decreases binding to SSTR4 and SSTR5) but at a similar level to that of peptides **4** and **5**.

## Discussion

Here we studied the effect of substituting Phe residues for Dfp (3-(3′,5′-difluorophenyl)-alanine) in the 14 amino acid structure of Somatostatin containing a D-Trp in position 8. All six peptides displayed well-dispersed 2D NMR data, which were used to characterize their main conformations in aqueous solution. It has been postulated that electron-deficient aromatic residues in position 11 destabilize the fold of the analog due to their limited capacity to shield the natural Phe6^25^. However, we found that the Somatostatin analog [D-Trp8,Dfp11]-SRIF (peptide **3)** showed a strong interaction between the aromatic rings of Phe6 and Dfp11, and remarkably high activity and selectivity towards SSTR2. Interestingly, when the Dfp residue was placed in position 7 (peptide **2)**, the analog displayed high affinity and selectivity for SSTR3.

**Figure 4:**
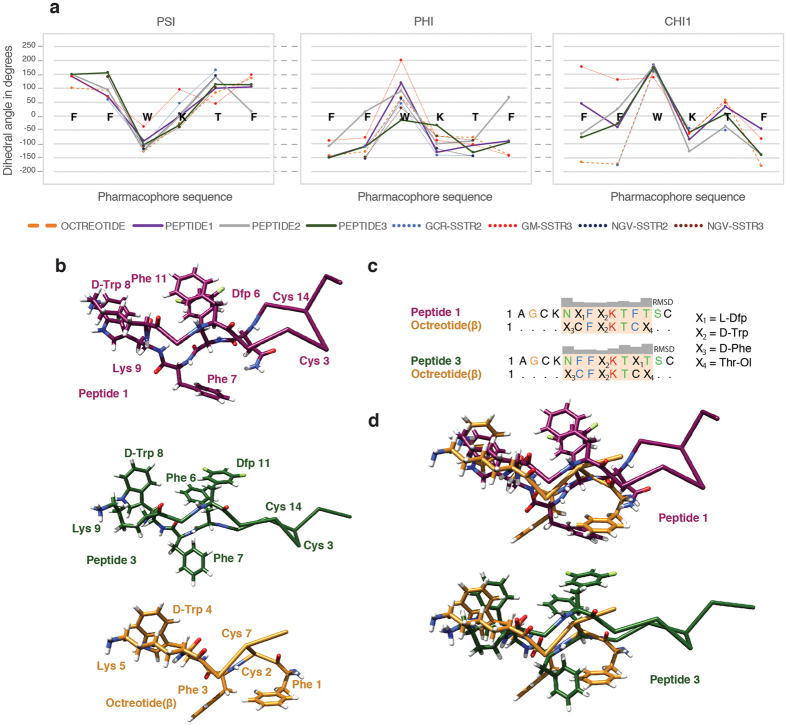
Comparison of representative angles and structures of some Somatostatin analogs. (**a**) Comparison of Φ, Ψ and **Χ**_1_ angles defining the pharmacophore region. The positions used for the analysis are represented with the amino acids corresponding to the native sequence of Somatostatin (-FFWKTF-), which are shown as a guideline. The plots show the dihedral angles of eight representative analogs (including peptides **1**, **2** and **3**) of SSTR2 and SSTR3 binders: **OCTR**: Octreotide, D-Phe,c[Cys,Phe,D-Trp,Lys,Thr,Cys],Thr-ol, (Melacini *et al*.^41^) **PEPT1**: peptide **1**, [L-Dfp6,D-Trp8]-SRIF), **PEPT2**: peptide **2**, [L-Dfp7,D-Trp8]-SRIF), **PEPT3**: peptide **3**, [D-Trp8,L-Dfp11]-SRIF), **GCR-SSTR2** (H_2_N-CO-D-Phe,c[Cys,Aph(CONHOCH_3_),D-Trp,Lys,Thr,Cys],Thr-NH_2_ (Grace *et al*.^17^), **GM-SSTR3** (c[Cys,Phe,Tyr,Agl,Lys,Thr,Phe,Cys] (Gairí *et al*.^16^), **NGV-SSTR2** and **NGV-SSTR3** (D-Phe,c[Cys,Phe,D-Trp,Lys,Thr,Cys],Thr-NH_2_) and D-Phe,c[Cys,Phe,D-Trp,Lys,Thr,Cys],Thr-NH_2_ (Nikiforovich *et al*.^18^). (**b**) The lowest energy structures of peptide **1** (receptor 2 and 3 binder) and peptide **3** (receptor 2 binder), shown in purple and green respectively, with the side chains of selected amino acids displayed and labeled. The structure of the minimized average beta-sheet structure of Octreotide (PDB ID: 1SOC, Melacini *et al*.^41^) is shown in gold. (**c**) Structural alignment of peptide **1** and **3** to Octreotide, showing the RMSD difference in arbitrary units (generated with Chimera). X_1_ = L-Dfp (L-3-(3′,5′-difluorophenyl)alanine), X_2_ = D-Trp, X_3_ = D-Phe, X_4_ = L-Thr(ol). (**d**) Superposition of the structures of peptide **1** (top) and **3** (bottom) with Octreotide.

Several studies using short-ring Somatostatin analogs have correlated the selectivity towards a given receptor type with the 3D conformations of the analogs^14^,^15^,^16^,^17^,^18^. For instance, the structure of Octreotide has been determined by X-ray^40^ and NMR^41^ and is considered one of the preferred conformations recognized by SSRT2^17^,^18^. NMR studies of other short-ring analogs revealed the presence of conformations preferentially recognized by SSTR1^15^, SSTR3^16^ and SSTR4^14^. Indeed, it is widely accepted that the geometry of the peptide backbone in the pharmacophore region plays an important role in receptor recognition^18^. In order to compare the similarities and differences of these new analogs to others previously characterized, we collected the structural information on SSTR2- and SSTR3-selective binders from the literature. The comparison focused on the psi (Ψ), phi (Φ), and chi1 (**Χ**_1_) angles in the pharmacophore and on five representative analogs selected on the basis of their binding preferences (Fig. 4a). The analysis also included peptides **1–3**, whose affinities for SSTR2 and SSTR3 are in the range of the Somatostatin hormone. As expected, since most of the analyzed conformers displayed a beta-turn structure, there was a high degree of similarity with respect to the Ψ and **Χ**_1_ values, whereas the variability was slightly larger for the Φ angle, especially in the case of the residue occupying the Tryptophan position.

In order to explore the similarities and differences of the pharmacophore region at the side-chain level, we superimposed the 3D structure of Octreotide (PDB ID 1SOC) on the structures of peptides **1** and **3 (**Fig. 4d). The comparison revealed a striking similarity, not only around the beta-turn area but also in the position of the aromatic rings, where the Phe1 and Phe3 of Octreotide are located in a similar position to Phe7 in the 14-residue analogs (and presumably in the natural hormone). These similarities could explain the high affinity of Octreotide and peptides **1** and **3** for SSTR2. However, these comparisons also revealed small differences at the backbone and at the side-chain levels. These differences with the Octreotide structure (Fig. 4b) were more significant for peptides **1** and **2**, which displayed similar affinity values for SSTR2 and SSTR3, than for peptide **3**, which showed greater affinity and selectivity towards SSTR2. Some of these differences may explain the preferential binding of peptides **1** and **2** to SSTR3.

In summary, here we have optimized an efficient method for preparing the Dfp amino acid with a 99% ee and designed, synthesized, and structurally characterized six new 14-residue Somatostatin analogs. Peptide **3**, [D-Trp8,Dfp11]-SRIF, had the highest activity and selectivity for SSTR2, the values being higher than those of Octreotide and similar to those of the natural hormone. In addition, peptide **1 (**[Dfp6,D-Trp8]-SRIF) and peptide **2 (**[Dfp7,D-Trp8]-SRIF), whose conformations in solution differ in their aromatic packing, both showed high activity towards SSTR2 and SSTR3. Our work indicates that Dfp amino acids are efficient building blocks in the synthesis of peptide analogs and opens up new possibilities for designing molecules with tuned selectivity and specificity towards SSTR2 and SSTR3.

## Methods

### Synthesis

Fmoc-Dfp-OH was prepared as shown in Fig. 2a. Experimental details can be found in the supporting information.

The synthesis of peptides **1–6** was performed by SPPS on a 2-Cl-Trt chloride resin (0.80–1.60 mmol/g) using the Fmoc/*t*Bu strategy (Fig. 2b). Initially, the first amino acid Fmoc-L-Cys(Trt)-OH (1 eq.) was coupled in the presence of DIPEA (4 eq.) in DCM as solvent for 40 min and finally end-capped with methanol (0.8 mL.g^−1^). Fmoc was then removed by treating the peptidyl resin with 20% piperidine in DMF (1 × 1′ and 1 × 5′). The second amino acid Fmoc-L-Ser(tBu)-OH (2.5 eq.) was coupled in DMF for 40–60 min using DIPCDI (2.5 eq.) and HOBt (2.5 eq.) as activating reagents. The Kaiser test was used to check coupling completion. This procedure was repeated for the following 11 Fmoc-protected amino acids and for the last Boc-L-Ala-OH. When Fmoc-L-Msa-OH or Fmoc-L-Dfp-OH was coupled, only 1.5 eq. were used. Cleavage of the fully protected linear peptide from the resin was carried out using a cleavage cocktail (DCM:TFE:AcOH, 70:20:10 (v/v)) for 2 h. The formation of the disulfide bridge in all the analogs was achieved using iodine at room temperature and quenching with 1N sodium thiosulfate. The aqueous layer was extracted with DCM (3 × 4 mL), the combined organic layer was washed with a mixture of an aqueous citric acid 5% solution/sodium chloride (1:1) and evaporated under reduced pressure. Finally, total deprotection of the side-chains was performed using an acidic mixture (TFA/DCM/Anisole/H2O) for 4 h. The remaining solution was then washed with heptane (20 mL), and the aqueous layer was precipitated in Et_2_O (−10 °C) to obtain Somatostatin analogs **1–6**.

### NMR and Computational methods

Spin systems and sequential assignments for all analogs were obtained from 2D TOCSY and NOESY homonuclear experiments, recorded at 285 K using a Bruker Avance III 600-MHz spectrometer equipped with a quadruple resonance z-gradient CryoProbe (5 mm QPQCI). All NMR data were processed with NMRPipe^42^ and Cara^43^ was used to facilitate the assignment of the spectra. Distance restraints derived from fully assigned peaks in NOESY experiments were used for structure calculation. The structures were calculated with the program CNS 1.2^37^. The calculation protocol consisted of an implicit water simulated-annealing of 120 structures using 8,000 cooling steps followed by an explicit water refinement of the calculated structures using all experimental restraints during 1200 steps. PyMOL^44^ and UCSF Chimera^45^ were used to visualize the structures and generate the figures. BMRB codes for peptides 1-6: 26794, 26795, 26796, 26707, 26798 and 26799.

## Additional Information

**How to cite this article**: Martín-Gago, P. *et al*. Peptide aromatic interactions modulated by fluorinated residues: Synthesis, structure and biological activity of Somatostatin analogs containing 3-(3′,5′-difluorophenyl)-alanine. *Sci. Rep*. **6**, 27285; doi: 10.1038/srep27285 (2016).

## Supplementary Material

Supplementary Information

## Figures and Tables

**Table 1:** *K*_i_ values (nM) of Somatostatin, [D-Trp8]-SRIF, Octreotide, and Somatostatin analogs to receptors SSTR1–5.

		SSTR1	SSTR2	SSTR3	SSTR4	SSTR5
Somatostatin (SRIF)		1.88	0.016	0.25	1.55	0.76
Octreotide		>300	0.770	13.0	>300	21.0
1	[L-Dfp6,D-Trp8]-SRIF	21.0	0.350	0.38	16.0	5.02
2	[L-Dfp7,D-Trp8]-SRIF	14.0	0.360	1.10	12.0	2.10
3	[D-Trp8,L-Dfp11]-SRIF	14.0	0.066	3.81	11.0	5.00
4	[L-Dfp6,L-Msa7,D-Trp8]-SRIF	38.0	0.170	5.46	150	82.0
5	[L-Msa7,D-Trp8.L-Dfp11]-SRIF	19.0	0.260	23.0	62.0	31.0
6	[L-Dfp6,11,L-Msa7,D-Trp8]-SRIF	55.0	0.310	12.0	63.0	45.0

Shaded cells represent data close to the Somatostatin values. The binding measurements were performed by Eurofins Panlabs Taiwan, Ltd.
