# Supplementary material for: Peptide aromatic interactions modulated by fluorinated residues: Synthesis, structure and biological activity of Somatostatin analogs containing 3-(3′,5′difluorophenyl)-alanine
Source: Sci Rep. 2016 Jun 7;6:27285. doi: 10.1038/srep27285 (PMC4895178; doi:10.1038/srep27285)
Supplement: Supplementary Information [file srep27285-s1.pdf]

Supporting Information

Peptide aromatic interactions modulated by fluorinated residues: Synthesis, structure and biological activity of Somatostatin analogs containing 3-(3',5'difluorophenyl)-alanine.

Pablo Martín-Gago,[1,#] Álvaro Rol,[1,#] Toni Todorovski,[1] Eric Aragón,[1] Pau Martin-Malpartida,[1] Xavier Verdaguer,[1,3] Mariona Vallès-Miret,[2] Jimena Fernández-Carneado,[2] Berta Ponsati,[2] Maria J. Macias[1,4,]* and Antoni Riera[1,3,]*

1 Institute for Research in Biomedicine (IRB Barcelona). The Barcelona Institute of Science and Technology. Baldiri Reixac, 10, Barcelona 08028, Spain; E-Mails: pablo.martin.gago@gmail.com (P.M.-G.); a.rol.rua@gmail.com (A.R.); toni.todorovski@irbbarcelona.org (T.T.) eric.aragon@irbbarcelona.org (E.A.); pau.martin@irbbarcelona.org (P.M.-M.); xavier.verdaguer@irbbarcelona.org (X.V.)

2 BCN Peptides S.A. Pol.Ind. Els Vinyets-Els Fogars, Sector II. Ctra. Comarcal 244, Km. 22, 08777 Sant Quintí de Mediona, Barcelona 08777, Spain; E-Mails: mvalles@bcnpeptides.com (MVM); jfernandez@bcnpeptides.com (J.F.-C.); bponsati@bcnpeptides.com (B.P.)

3 Departament de Química Orgànica, Universitat de Barcelona, Martí i Franqués, 1-11, Barcelona 08028, Spain

4 Institució Catalana de Recerca i Estudis Avançats (ICREA), Passeig Lluís Companys, 23, Barcelona 08010, Spain

* Authors to whom correspondence should be addressed;
E-Mails: maria.macias@irbbarcelona.org (M.J.M.); antoni.riera@irbbarcelona.org (A.R.); Tel. +34-934-047-093 (A.R.); Fax: +34-934-047-095 (A.R). Tel.: +34-934-037-189 (M.J.M.);

General methods and instrumentation for non-natural amino acids synthesis: All reactions were carried out under nitrogen atmosphere unless otherwise specified. When dry solvents were necessary (dichloromethane, diethyl ether and tetrahydrofuran), the Innovative Technology Inc. Puresolv purification system Solvent Purification System (SPS) were used. Other dry solvents were purchased from Sigma-Aldrich and used without further purification.

All experiments were monitored by analytical thin layer chromatography (TLC) performed on silica gel TLC-aluminum sheets (Merck 60 F254). Chromatographic purifications were carried out using a CombiflashR (Teledyne Isco) automated chromatography system unless otherwise stated. Silica gel Redi*Hep*R columns were used. The elution was carried out using hexanes/EtOAc gradients.

NMR spectra of small molecules (non peptidic) were recorded at room temperature on a Varian Mercury 400. 1H NMR and 13C NMR spectra were referenced to residual solvent peaks. 19F NMR spectra were referenced by the spectrometer without external reference. Signal multiplicities in the 13C spectra have been assigned by DEPT (Direct Enhancement by Polarization Transfer) and HSQC (Hetero-nuclear Single Quantum Correlation) experiments and are described as C (quaternary), CH (tertiary), CH2 (secondary) and CH3 (primary). The following abbreviations were used to define the multiplicities: s, singlet; d, doublet; t, triplet; q, quartet; m, multiplet; br, broad. The coupling constants (*J*) are measured in hertz (Hz).

Melting points were measured using DSC 822e Mettler-Toledo apparatus. HRMS experiments were carried out in The Mass Spectrometry Core Facility located in the Institute for Research in Biomedicine of the using NanoESI techniques. CHNS elemental analyses have all been determined by the *Unitat de Tècniques Separitives i Síntesi de Pèptids* (The Separation Techniques and Peptide Synthesis Unit) located at the Barcelona Science Park. All IR spectrums have been obtained using a Thermo Nicolet Nexus FT-IR Fourier transform spectrometer. The samples were prepared by either dissolution in solvent and subsequent

formation of a film on a NaCl disc by evaporation of the prepared solution or by the preparation of a KBr disc.

Optical rotations were measured at room temperature (25°C) using a Jasco P-2000 iRM-800 polarimeter. A cell with a length of 1 dm and a volume of 1 mL has been used. The concentration is expressed in the form g/100 mL. A sodium lamp with a wavelength of 589 nm has been employed.

(Z)-4-(3,5-Difluorobenzylidene)-2-methyloxazol-5(4H)-one, (1)

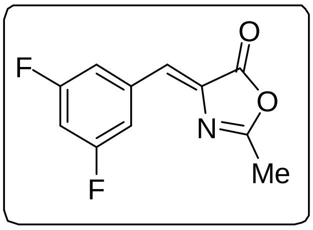

To a two-neck 100-mL round-bottom flask fitted with a mechanical stirrer and a condenser, under N_2 atmosphere, was added N-acetylglycine (8.2 g, 70 mmol), Ac_2O (18 mL, 175 mmol), NaOAc (5.8 g, 70 mmol) and 3,5-difluorobenzaldehyde (10 g, 70 mmol). The mixture was stirred for 2 h at 100 ºC to provide a light brown solution. The crude was allowed to cool to room temperature and a stirrable paste was formed. Then, it was cooled to 0 °C and cold water (60 mL) was added. The mixture was stirred 10 min. Filtration, washing the collected solid with cold water (3x15 mL) and drying at 50 °C in vacuo provided 14.1 g (90% yield) of the titled compound **(1)** as a light brown powder, which was used in the next reaction without further purification.

Mp: 155-158 °C. **IR** (KBr): ν_{max} 3051, 1800, 1665 cm^{-1}. **^{1}H-NMR** (400 MHz, $CDCl_3$): δ 7.6 (m, 2H, Ar), 7.0 (s, 1H, CH), 6.9 ((tt, J = 9 and 2 Hz, 1H), 1H, Ar), 2.4 (s, 3H, CH_3) ppm. **^{13}C-NMR** (100 MHz, $CDCl_3$): δ 167.6 (CO), 167.0 (C, Az), 162.9 ((dd, J_F = 249, 13 Hz) 2 C, Cε Ar), 135.8 ((t, J_F = 10 Hz) C, Ar), 134.6 (s, C, Az), 128.1 ((t, J_F = 3 Hz) CH, Cβ), 114.5 (m, 2 CH, Cδ Ar), 106.7 ((t, J_F = 26 Hz) 2 CH, Cz Ar), 15.7 (CH_3) ppm. **^{19}F NMR** (376 MHz, $CDCl_3$) δ = -118.4 (t, J= 7 Hz, 2F) ppm. **HRMS**: calcd. for $C_{11}H_7F_2NO_2$: 223.0445; $(M+H)^+$ found, 224.0519.

(Z)-Methyl 2-acetamido-3-(3,5-difluorophenyl)acrylate, (2)

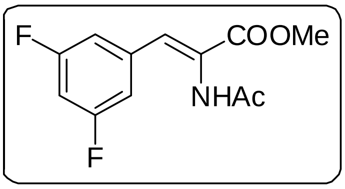

(Z)-4-(3,5-Difluorobenzylidene)-2-methyloxazol-5(4H)-one **(1)** (10 g, 45 mmol, 1 eq) was dissolved in anhydrous MeOH (133 mL) in a two-neck 250-mL round-bottom flask fitted with a mechanical stirrer and a condenser, and under N_2 atmosphere. MeONa (3.8 g, 49 mmol, 1.1 eq) was added dropwise, and the reaction mixture was stirred for 2 h at 70 ºC. The crude was allowed to cool to room temperature and the solvent was evaporated under reduced pressure. The crude mixture was redissolved, neutralized with HCl and extracted with AcOEt. The combined extracts were washed with brine solution and dried over $MgSO_4$. After filtration of $MgSO_4$, the solvent was removed under vacuum and the brown colored residue was purified by silica gel flash chromatography to give pure 6.9 g (27 mmol, 65 % yield) of the titled product **(2)** as a white solid.

Mp: 176-178 °C. **IR** (KBr): ν_{max} 3261(b), 1719, 1663, 1284 cm^{-1}. **^{1}H-NMR** (400 MHz, $CDCl_3$): δ 7.28 (s, 1H, CHβ), 7.09 (s, 1H, NH), 6.96 (d, J = 5 Hz, 2H, Ar), 6.77 (t, J = 9 Hz, 1H, Ar), 3.87 (s, 3H, -OCH3), 2.15 (s, 3H, -C(O)CH3) ppm. **^{13}C-NMR** (100 MHz, $CDCl_3$): δ 168.4 (CO), 165.2 (CO), 162.8 ((dd, J_F = 248, 13 Hz) 2 CH, CHε Ar), 137.1 ((t, J_F = 9 Hz), C, Ar), 128.9 (CH, Cβ), 125.5 (C, Cα), 110.1 (m, 2 CH, Cδ Ar), 104.5 ((t, J_F = 25 Hz), CH, Cz Ar), 53.0 (CH_3, MeO), 23.5 (CH_3, Ac) ppm. **^{19}F NMR** (376 MHz, $CDCl_3$) δ = -110.6 (t, J= 8 Hz, 2F) ppm. **HRMS**: calcd. for $C_{12}H_{11}F_2NO_3$: 255.0707; $(M+H)^+$ found, 256.0781.

(S)-Methyl 2-acetamido-3-(3,5-difluorophenyl)propanoate, (3)

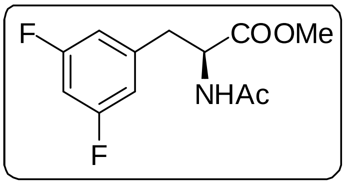

A high pressure reaction vessel was charged with a mixture of (Z)-methyl 2-acetamido-3-(3,5-difluorophenyl)acrylate **(2)** (7.35 g, 32.7 mmol), (S,S)-[(COD)-MaxPHOS] Rh(I) cat (3%) and degassed MeOH (70 mL). The reactor was purged with nitrogen-vacuum cycles and

subsequently, with hydrogen. Finally, the reaction vessel was pressurized to 5 bar with H_2 and stirred at room temperature for 20 h. The mixture was vented with nitrogen and the solvent was removed at reduced pressure. The resulted mixture was redisolved in AcOEt and the catalyst was removed by filtration of the crude in a silica column. After elimination of the solvent at reduced pressure, the (S)-methyl 2-acetamido-3-mesitylpropanoate **(3)** was obtained as a white pure solid in 99% yield and 99% enantiomeric excess.

$[\alpha]_D$= +156.2 (c 0.50, $CHCl_3$). **Mp:** 113-117 °C. **IR** (KBr): ν_{max} 3246(b), 2994, 1719, 1661 cm^{-1}. **^{1}H-NMR** (400 MHz, $CDCl_3$): 6.71 (m, 1H, CH Ar), 6.63 (m, 2H, CH Ar), 5.96 (d, J = 6 Hz, 1H, NH), 4.87 (dt, J = 6 and 8 Hz, 1H, CHα), 3.76 (s, 3H, $-OCH_3$), 3.12 (ddd, J = 6, 14 and 35 Hz, 2H, CH_2β), 2.02 (s, 3H, $-C(O)CH_3$) ppm. **^{13}C-NMR** (100 MHz, $CDCl_3$): δ 171.5 (CO), 169.6 (CO), 162.9 ((dd, J_F = 249, 13 Hz) 2 C, Cε Ar), 139.9 ((t, J_F = 9 Hz) C, Ar), 112.1 (m, 2 CH, Cδ Ar), 102.9 ((t, J_F = 25 Hz) CH, Cz Ar), 52.9 (CH, Cα), 52.5 (CH_3, MeO), 37.6 (CH_2, Cβ), 23.1 (CH_3, Ac) ppm. **^{19}F NMR** (376 MHz, $CDCl_3$) δ = -110.10 (t, J= 8 Hz, 2F) ppm. **HRMS**: calcd. for $C_{12}H_{13}F_2NO_3$: 257.0863; $(M+H)^+$ found, 258.0937. **HPLC** (CHIRALPAK-IA, heptane/IPA 90:10, 1 mL/min, λ=254 nm, $t_R(R)$= 9 min, $t_R(S)$=11 min).

L-3-(3,5-difluorophenyl)-alanine hydrochloride, (4)

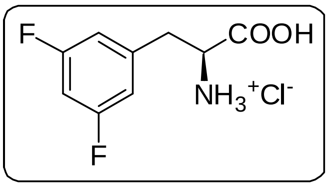

A mixture of (S)-methyl 2-acetamido-3-(3,5-difluorophenyl) propanoate **(3)** (7.0 g, 27.2 mmol) and concentrated HCl (36 mL) was subjected to reflux conditions for 30 minutes. Then, 77 mL of HCl (6M) was added, and the reaction was stirred for 6 h at the same temperature. After the reaction vessel spontaneously get the room temperature, the solvent is evaporated at reduced pressure and the white solid obtained was filtrated, washed and dried to quantitatively afford the hydrochloric salt of the L-3-(3,5-difluorophenyl)alanine **(4)**.

[α]D= +11.3 (*c* 0.50, H_2O). **Mp:** 215-219 °C. **^{1}H-NMR** (400 MHz, CD_3OD) δ 7.06 – 6.79 (m, 3H, CH Ar), 4.29 (dd, *J* = 8 and 6 Hz, 1H CHα), 3.33 (m, 1H, CH_2β), 3.16 (dd, *J* = 8 and 15 Hz, 1H, CH_2β) ppm. **^{13}C-NMR** (100 MHz, CD_3OD): *δ* 169.3 (CO), 164.5 (d, *J* = 13 Hz, C, Cε Ar), 162.1 (d, *J* = 13 Hz, C, Cε Ar), 138.6 (C, Cγ Ar), 112.2 (m, 2C, Cδ Ar), 102.67 (t, *J* = 26 Hz, C, Cz Ar), 53.2 (C, Cα), 35.33 (C, Cβ) ppm. **^{19}F NMR** (376 MHz, $CDCl_3$) δ -111.5 (t, *J* = 8 Hz, 2F) ppm.

N-Fmoc-L-3-(3,5-difluorophenyl)-alanine, (5)

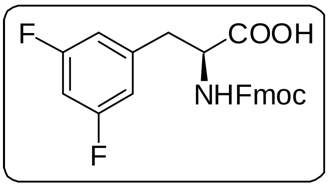

The L-3-(3,5-difluorophenyl)-alanine hydrochloride **(5)** (6.2 g, 26.2 mmol) was suspended in 66 mL of an aqueous solution of Na_2CO_3 (7.6 g, 72 mmol). The mixture was cooled to 0 ºC and a solution of FmocOSu (12.1 g, 36 mmol) in acetone (66 mL) was slowly added. The resulting mixture was allowed to warm to room temperature and stirred for 20h. Then, 20mL of water was added and the reaction mixture was extracted with ethyl acetate. The organic layer was back extracted with water, and the combined aqueous layers were washed with AcOEt, acidified to a pH of 1 with aqueous HCl and extracted with AcOEt. The combined organic layers were dried and concentrated *in vacuo* to give 9.9 g of N-Fmoc-L-3-(3,5-difluorophenyl)-alanine **V** as a white solid (89% yield, 99% e.e.). The resulting product can be purified by flash chromatography if necessary using mixtures AcOEt/Hexanes (1% AcOH).

[α]D= -139.1 (c 0.50, $CHCl_3$). **Mp:** 151-155 °C. **IR** (film): ν_{max} 3320, 3072, 1734, 1652 cm^{-1}. **^{1}H-NMR** (400 MHz, $CDCl_3$) δ 7.77 (d, J = 8 Hz, 2H, CH (Fmoc)), 7.56 (t, J = 7 Hz, 2H, CH (Fmoc)), 7.40 (t, J = 7 Hz, 2H, CH (Fmoc)), 7.35 – 7.28 (m, 2H, CH (Fmoc)), 6.70 (m, 3H, Ar), 5.26 (d, J = 8 Hz, 1H, NH), 4.69 (dd, J = 6 and 13 Hz, 1H, CHα), 4.44 (dt, J = 10 and 17

Hz, 2H, CH_2 (Fmoc)), 4.22 (t, J = 7 Hz, 1H, CH (Fmoc)), 3.14 (ddd, J = 6, 14 and 39 Hz, 2H, $CH_2\beta$). **^{13}C-NMR** (100 MHz, CDCl3): d 174.7 (C, CO), 155.9 (C, CO), 143.7 (2C, Fmoc), 141.6 (2C, Fmoc), 139.6 (m, C, Ar), 128.0 (2CH, Fmoc), 127.3 (2CH Fmoc), 127.2 (2CH Fmoc), 125.2 (d, *JF* = 8 Hz, 2C, Ar), 120.3 (2 CH, Fmoc), 115.5 (m, 2C, Ar), 103.1 (t, *JF* = 25 Hz, CH, Ar), 67.4 (CH2, Fmoc), 54.4 (CH, Cα), 47.4 (CH, Fmoc), 37.8 (CH2, Cβ) ppm. **^{19}F-NMR** (376 MHz, $CDCl_3$) δ -109.86 (t, J = 7 Hz, 2F) ppm. **HPLC** CHIRALPAK-IA. Heptane/IPA-0.1% TFA 90:10, 0.5 mL/min, λ=254 nm, t_R(*D*)=12 min, t_R (*L*) =13 min.

(1-4) and Fmoc-L-Dfp-OH spectra

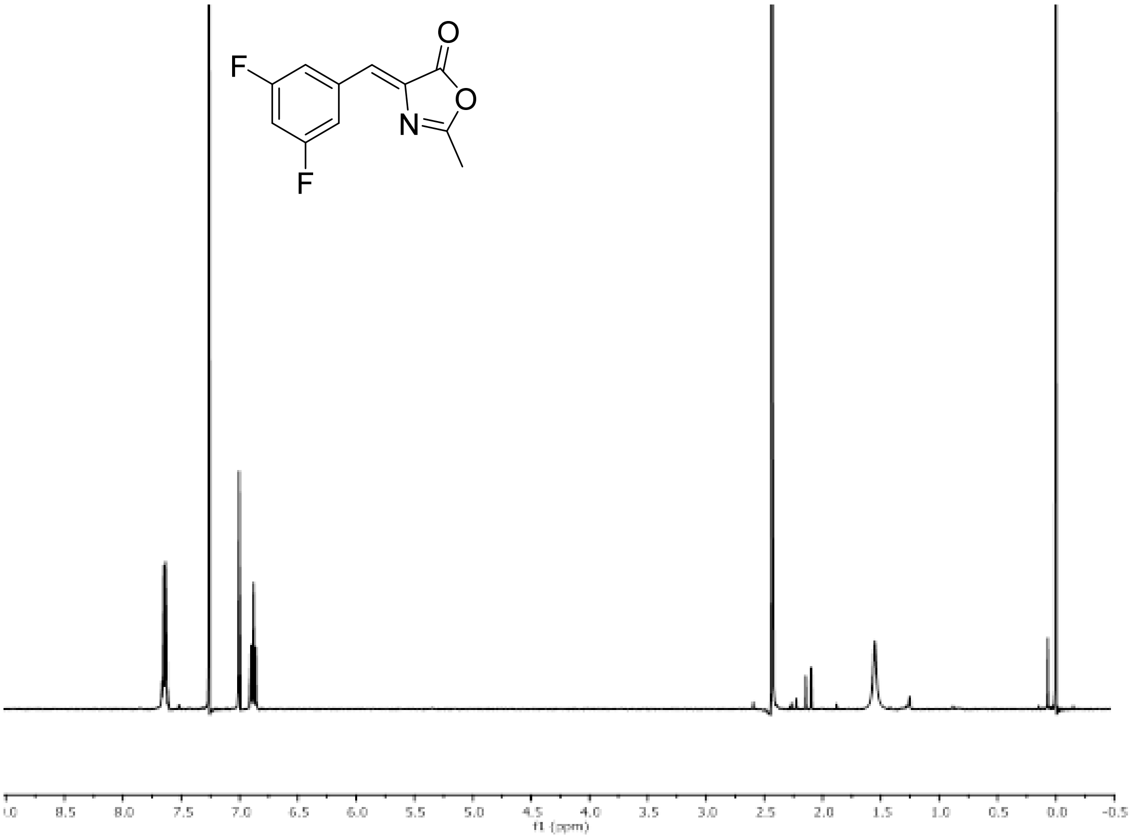

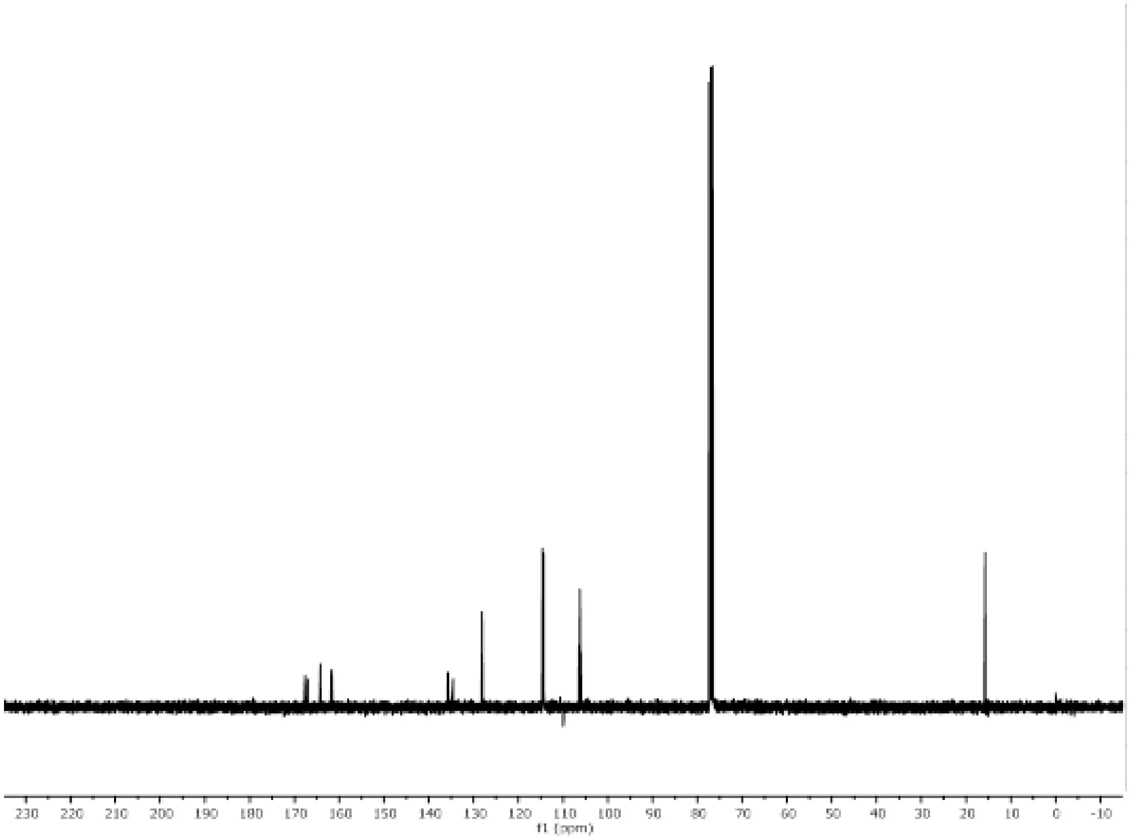

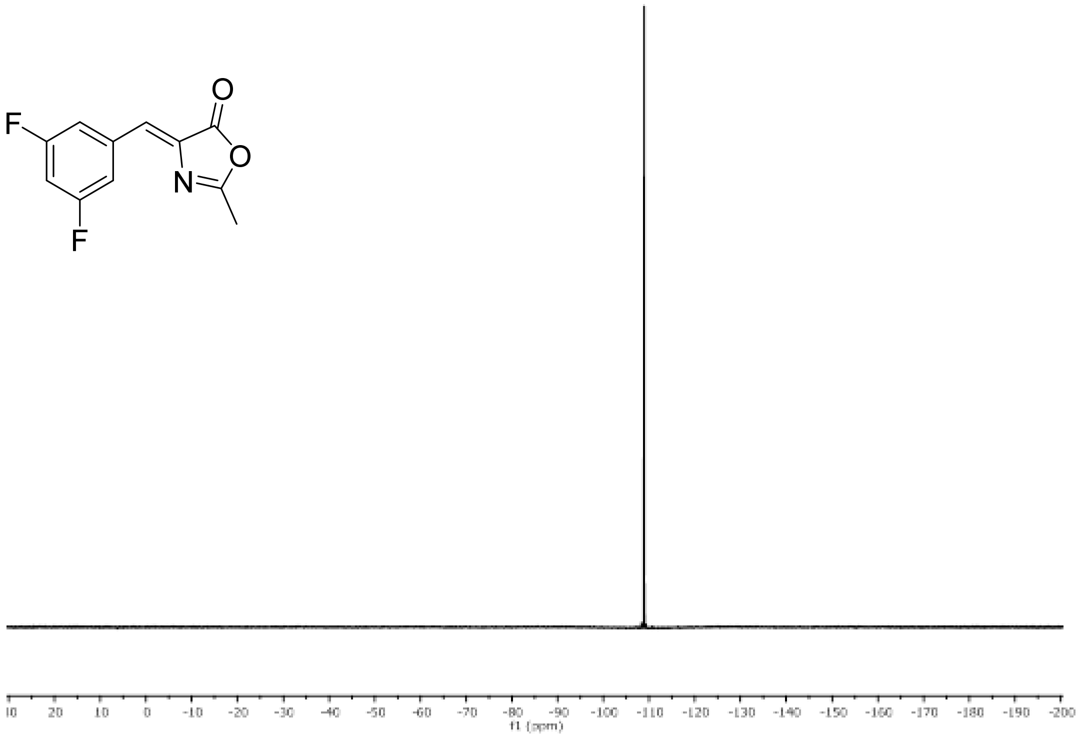
F
F
O
O
N
20 10 0 -10 -20 -30 -40 -50 -60 -70 -80 -90 -100 -110 -120 -130 -140 -150 -160 -170 -180 -190 -200
f1 (ppm)

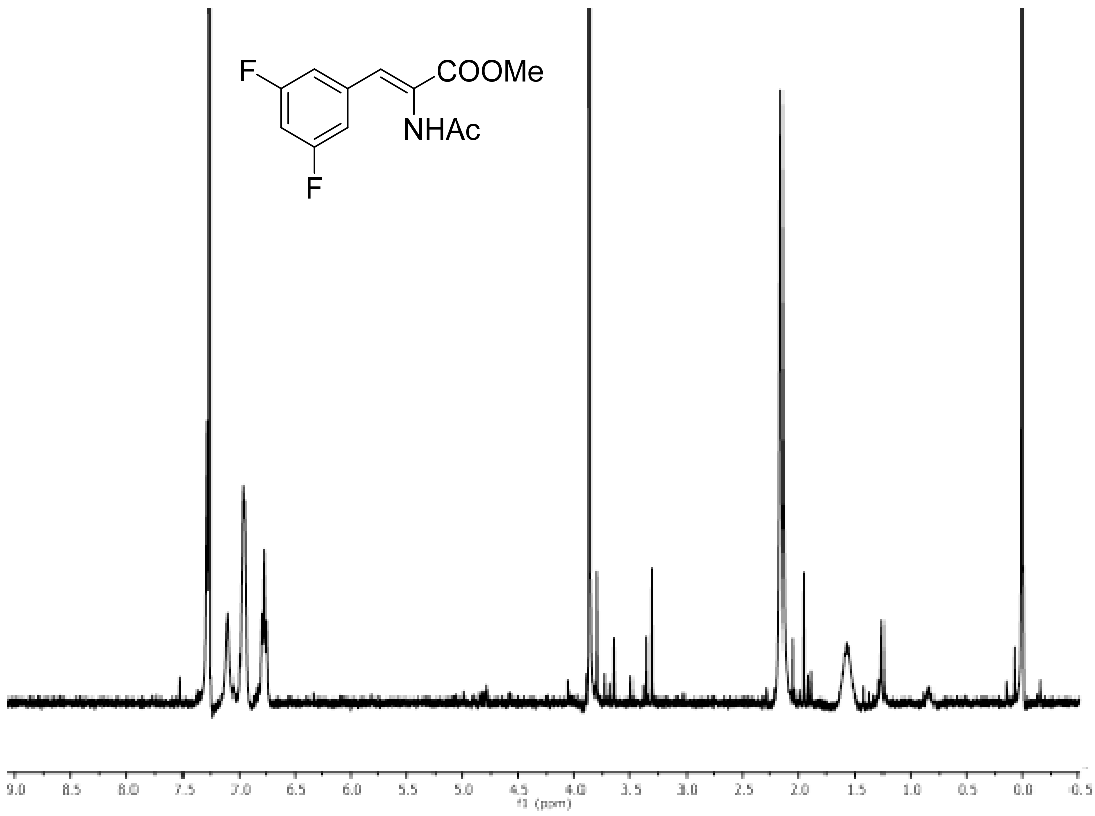
F
COOMe
NHAc
F
f1 (ppm)

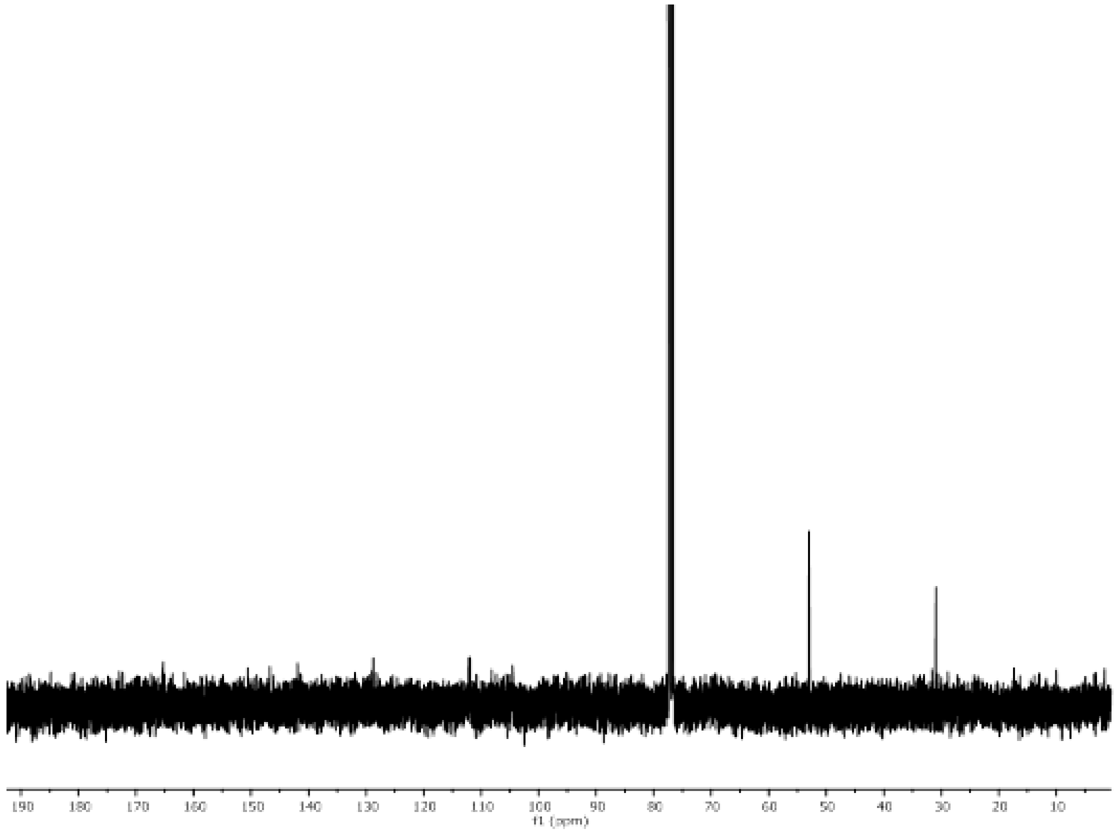
f1 (ppm)

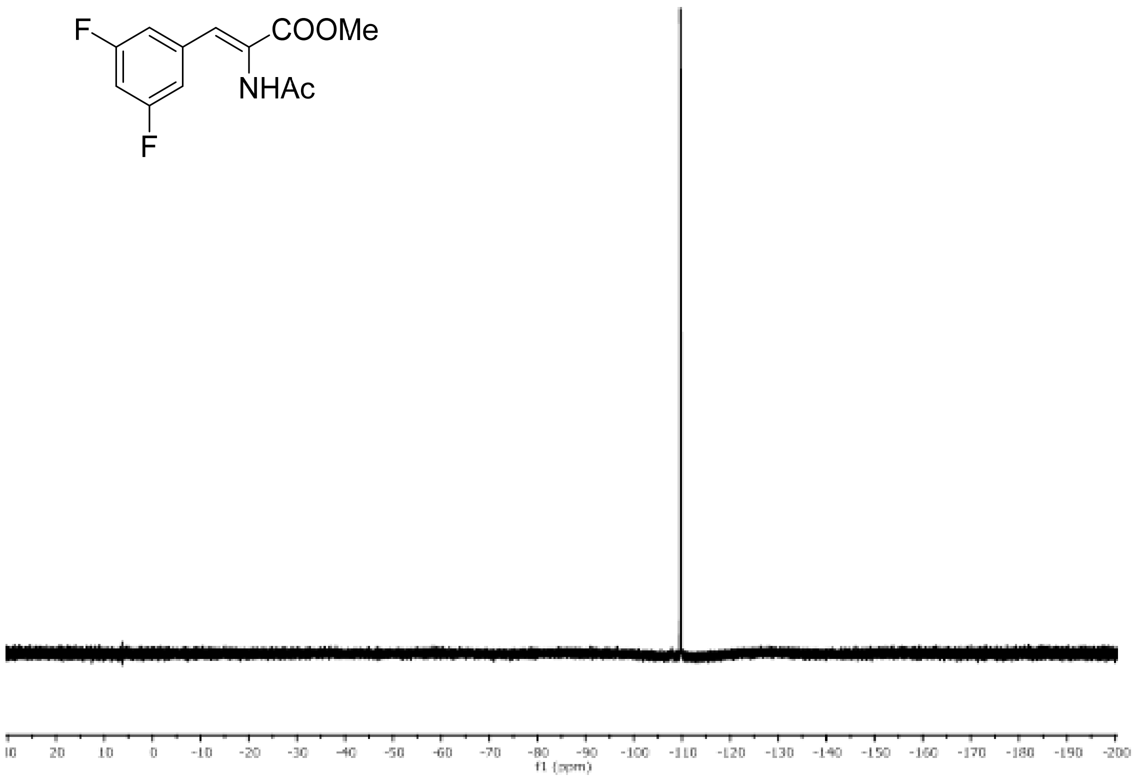
F
COOMe
NHAc
F
20 10 0 -10 -20 -30 -40 -50 -60 -70 -80 -90 -100 -110 -120 -130 -140 -150 -160 -170 -180 -190 -200
f1 (ppm)

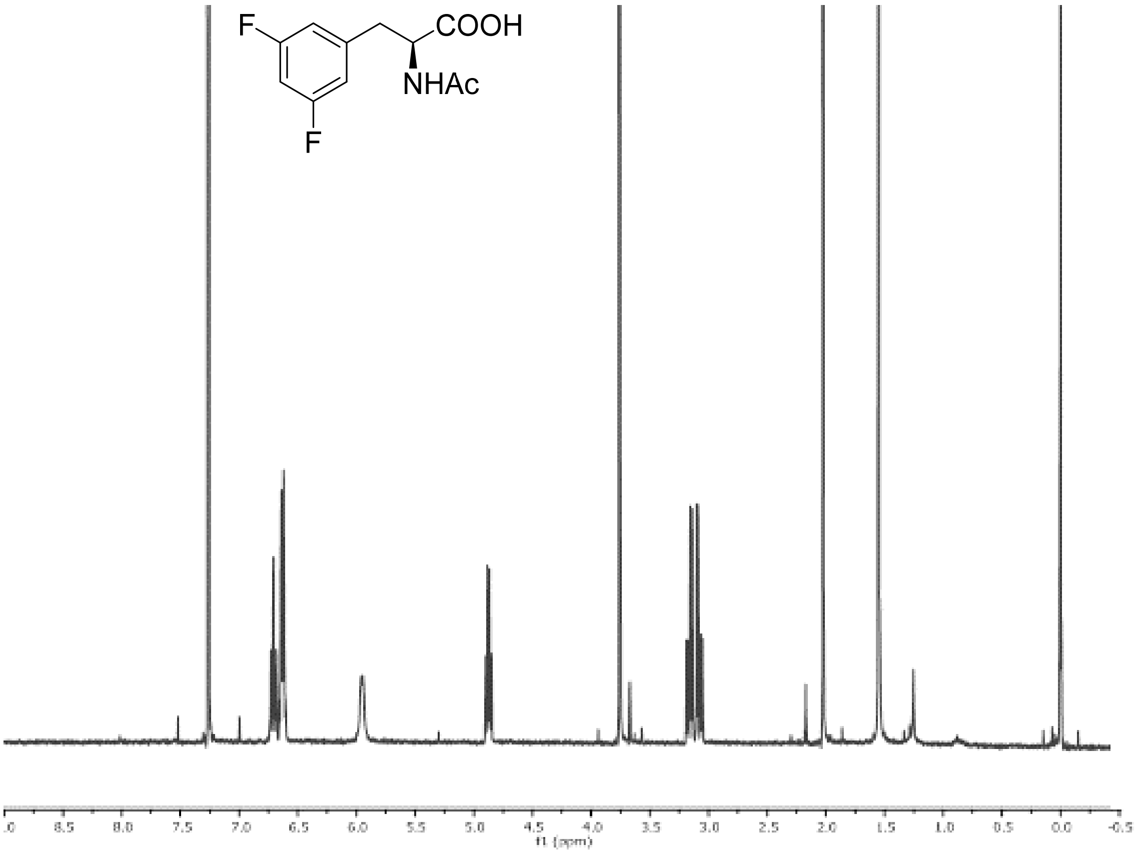
F
COOH
NHAc
F
f1 (ppm)

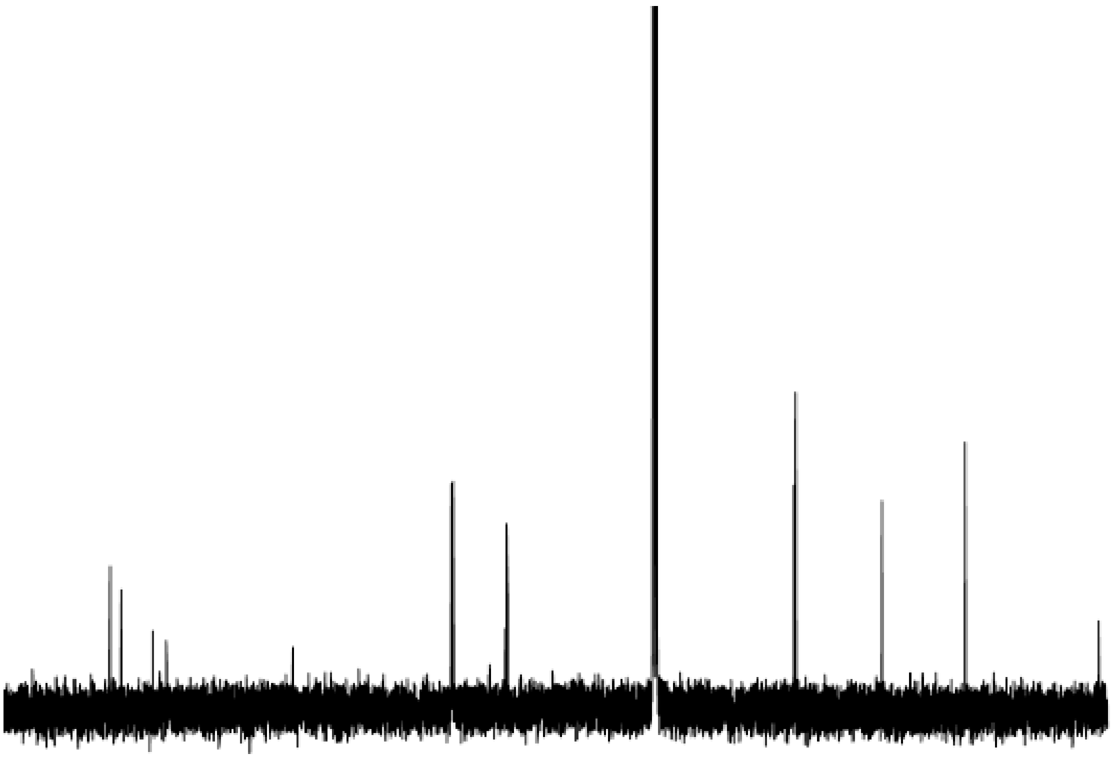
f1 (ppm)

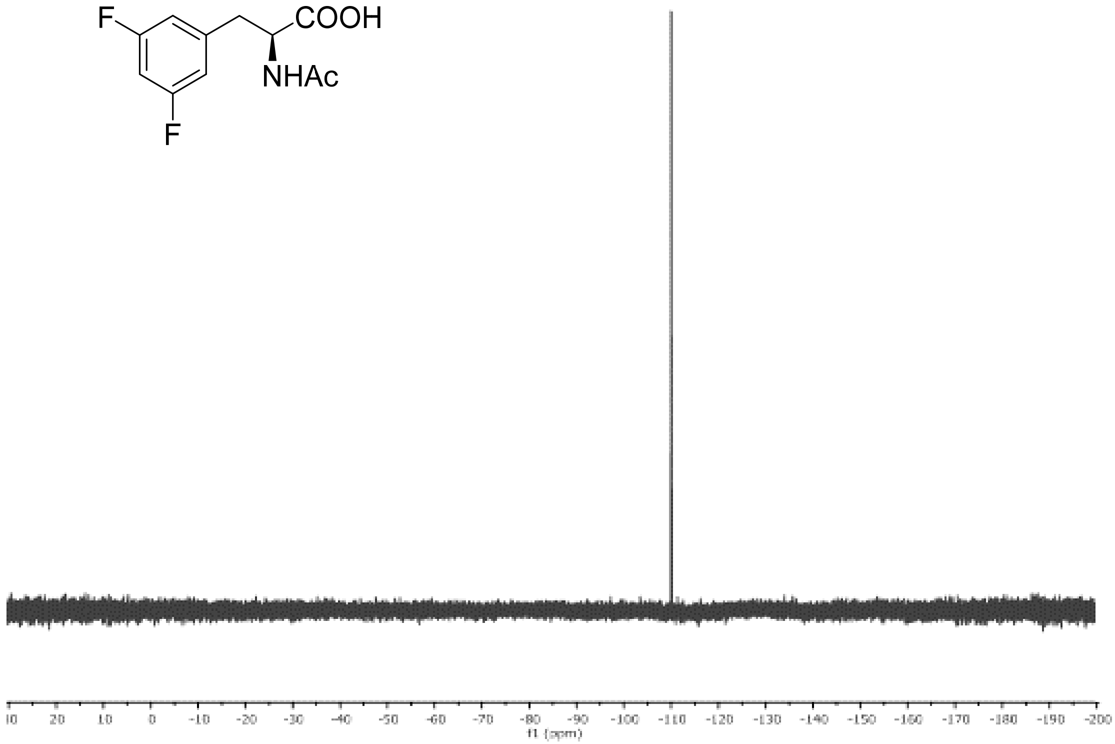
F
COOH
NHAc
F
f1 (ppm)

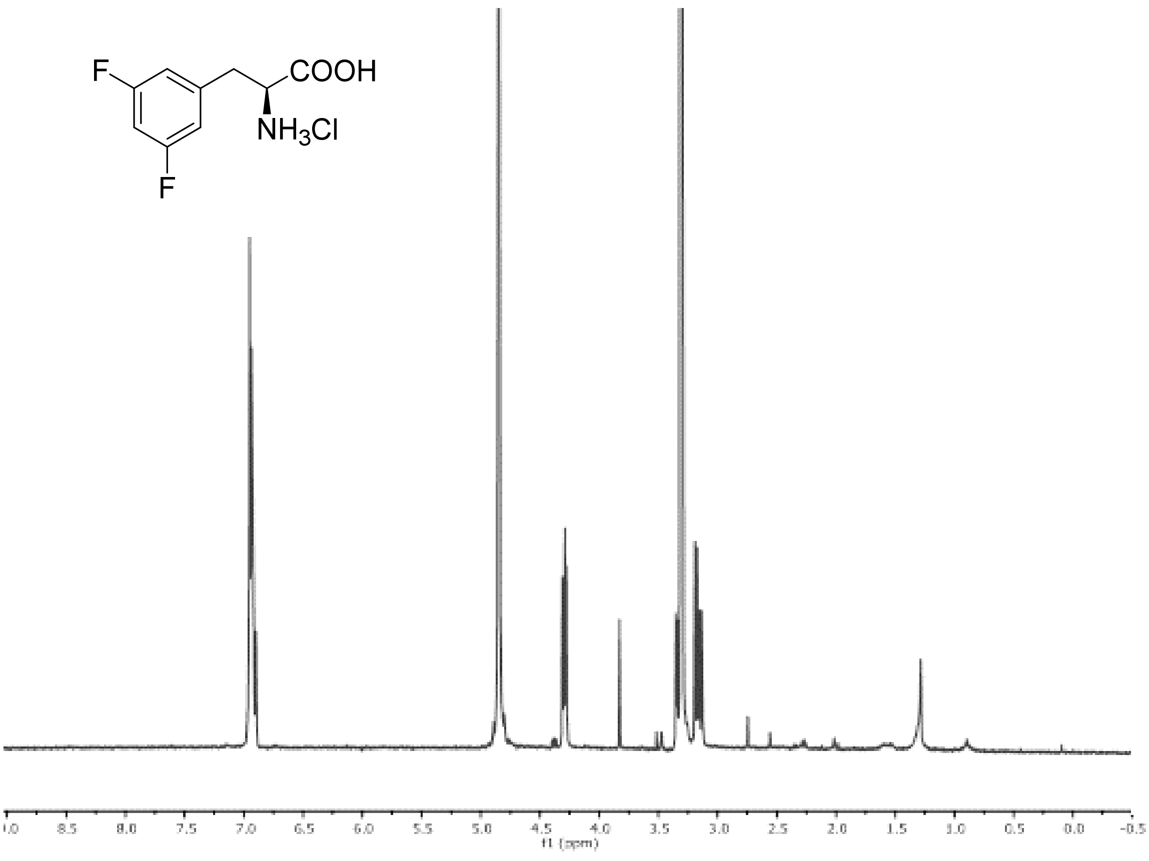
COOH
F
NH3Cl
F
f1 (ppm)

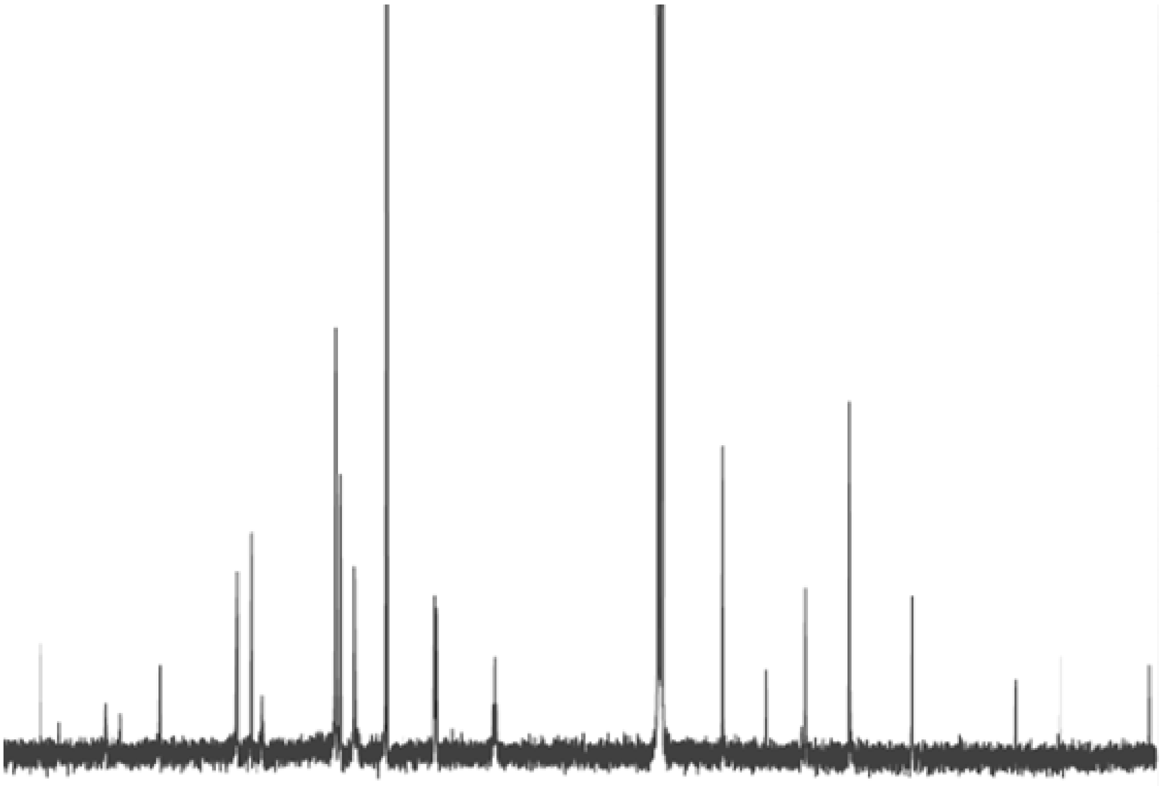
f1 (ppm)

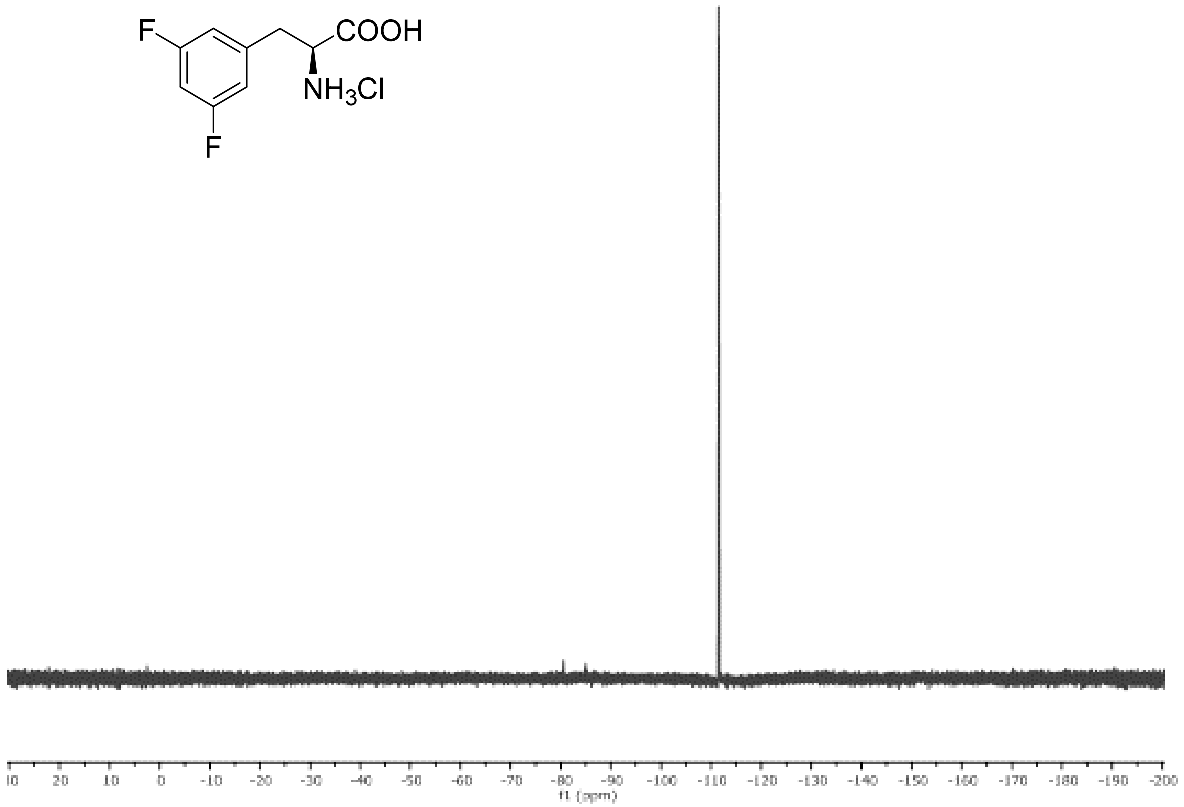
F
COOH
NH3Cl
F
20 10 0 -10 -20 -30 -40 -50 -60 -70 -80 -90 -100 -110 -120 -130 -140 -150 -160 -170 -180 -190 -200
f1 (ppm)

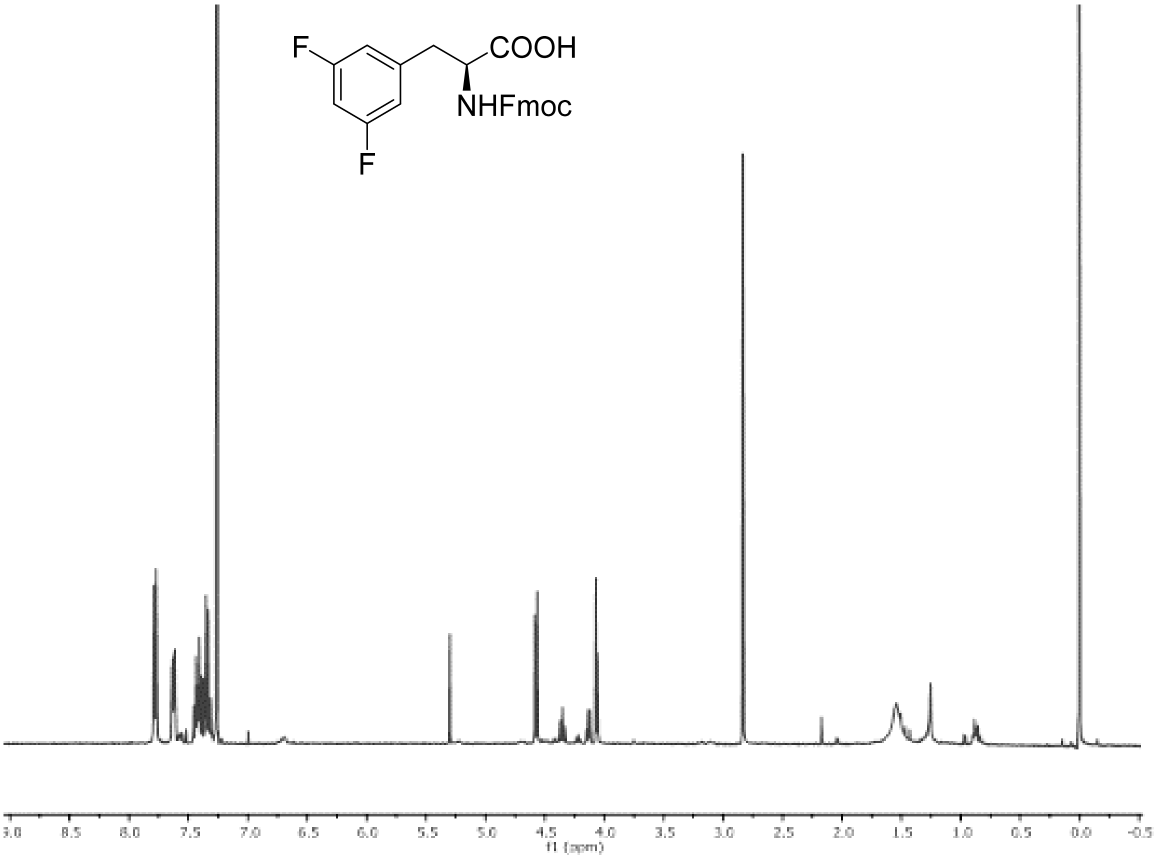
F
COOH
NHFmoc
F
9.0 8.5 8.0 7.5 7.0 6.5 6.0 5.5 5.0 4.5 4.0 3.5 3.0 2.5 2.0 1.5 1.0 0.5 0.0 -0.5
f1 (ppm)

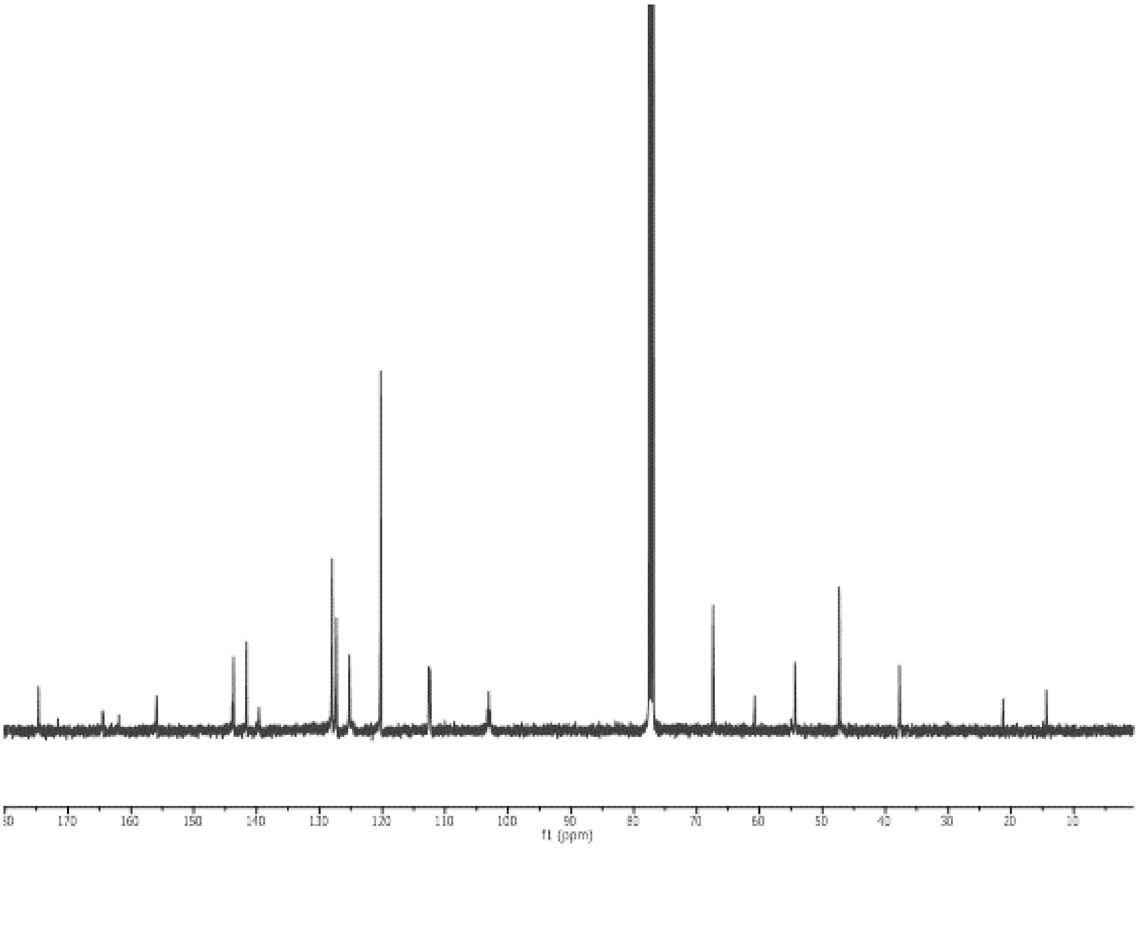
170 160 150 140 130 120 110 100 90 80 70 60 50 40 30 20 10
f1 (ppm)

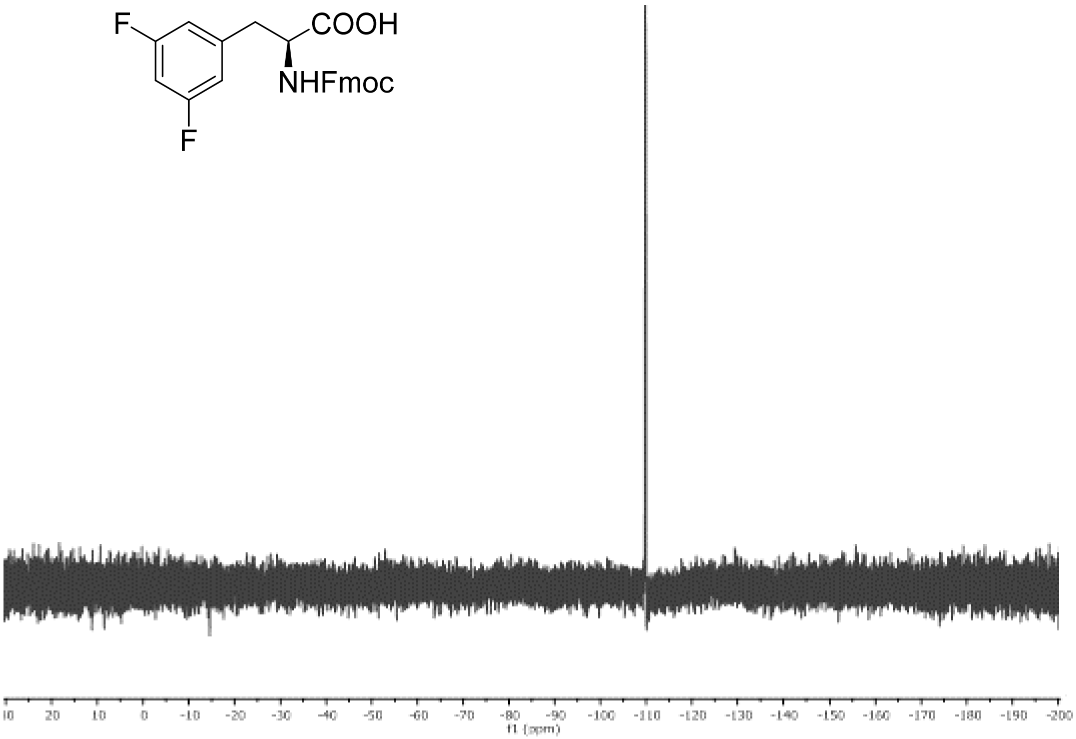
F
F
COOH
NHFmoc
20 10 0 -10 -20 -30 -40 -50 -60 -70 -80 -90 -100 -110 -120 -130 -140 -150 -160 -170 -180 -190 -200
f1 (ppm)

General procedures for peptide synthesis: All peptides were synthesized by SPPS using the Fmoc/tBu strategy. Solid-phase peptide syntheses were performed in polypropylene syringes provided with a porous polyethylene filter and attached to a vacuum manifold. The syringes volumes used were 5, 10 or 20 mL, depending on the scale of the synthesis. Reagents and solvents were added to the syringe containing the resin and the mixture was stirred in an orbital shaker. After each treatment, the solvent and the excess of reagents were removed by filtration through the vacuum system. After each synthetic step, the resin was washed with DCM (3 x 1 min), DMF (3 x 1 min) and DCM (3 x 1 min) unless otherwise specified.

Conventional couplings during the elongation of the peptide in solid phase were monitored by the "ninhydrin test". This colorimetric test allows the detection of primary amine groups on the resin. To perform the Kaiser or ninhydryn test,[1] the peptidyl-resin was washed with appropriate solvents and dried. A small amount of peptidyl-resin (0.5-2 mg) was transferred to a small glass tube. To this tube were added 6 drops of the reagent solution A and 3 drops of the reagent solution B. The mixture was heated at 100 °C for 3 min. The formation of a blue color on the beads or the supernatant is indicative of the presence of free primary amines (positive test) and thus of an uncompleted coupling. Conversely, a yellow coloration indicates the absence of free primary amines (negative test). The method is highly sensitive and a negative test ensures amino acid incorporation higher than 99.5%.

-Preparation of reagent solution A: Phenol (40 g) was dissolved in EtOH (10 mL) and the mixture was heated until complete dissolution of the phenol. An aqueous solution (20 mL, 10 mM) of KCN (65 mg in 100 mL of H2O) was added to freshly distilled

[1] Kaiser, E.; Colescott, R. L.; Bossinger, C. D.; Cook, P. I. *Anal. Biochem.* **1970**, *34,* 595-598.

pyridine (100 mL). Both solutions were stirred for 45 min with 40 g of Amberlite MB-3 ion exchange resin, filtered and combined.

-Preparation of reagent solution B: Ninhydryn (2.5 g) was dissolved in EtOH (50 mL). The resulting solution was kept in a flask protected from light.

All commercial amino acid were purchased from Senn Chemicals. The 3-mesityl alanine amino acid (Fmoc-L-Msa-OH) was synthesized as we previously reported.[2] HOBt and DIPCDI were purchased from SDS and SAF respectively. SRIF and octreotide were obtained from BCN Peptides S.A All other reagents were purchased from Aldrich unless otherwise noted and used without further purification. Chemical shifts are recorded in ppm. Analytical and preparative RP-HPLC were carried out with C8 Kromasil columns using gradients of solvent A (0.1% TFA in H2O) and solvent B (0.07% TFA in ACN). Room temperature (RT) is defined as 25 °C.

2D TOCSY and NOESY homonuclear experiments were acquired in a Bruker Avance III spectrometer (600 MHz).

---

[2] P. Martín-Gago, M. Gómez-Caminals, R. Ramón, X. Verdaguer, P. Martín- Malpartida, E. Aragón, J. Fernández-Carneado, B. Ponsati, P. López-Ruiz, M. Alicia Cortés, B. Colás, M. J. Macías, A. Riera, *Angew. Chem. Int.*, **2012**, *51*, 1820 –1825.

[L-Dfp6,D-Trp8]-SRIF (1): Somatostatin analog **1** was synthesized following the general procedure from 0.20 g of 2-Cl-Trt resin (0.8 mmol/g) and using Fmoc-L-Dfp-OH, affording 0.08 g in 43% yield (99% purity after purification). HPLC: tR = 16.5 [Gradient 25-60% B in 20 min, flux: 1 mL.min-1, λ=220 nm]. HRMS: calcd. for $C_{76}H_{102}F_2N_{18}O_{19}S_2$: 1672.6978; found, 1672.6981.

	**HN**	**Hα**	**Hβ**	**Hγ**	**Hδ**	**Hε**	**Hζ**	**Hη**
**1 Ala**	7.90	3.89	1.29	-	-	-	-	-
**2 Gly**	8.47	3.74	-	-	-	-	-	-
**3 Cys**	8.27	4.38	2.90 (β2)<br>2.72 (β3)	-	-	-	-	-
**4 Lys**	8.43	4.34	1.40	1.10 (γ2)<br>1.00 (γ3)	1.23 (δ2)<br>1.17 (δ3)	2.54	7.21	-
**5 Asn**	8.31	4.57	2.42	-	7.33 (δ21)<br>6.73 (δ22)	-	-	-
**6 Dfp**	8.28	4.49	2.72 (β2)<br>2.59 (β3)	-	6.29	-	6.48	-
**7 Phe**	8.03	4.30	2.71	-	6.91	7.11	7.06	-
**8 DTrp**	8.29	4.19	2.81 (β2)<br>2.70 (β3)	-	6.87	9.98 (ε1)<br>7.34 (ε3)	7.24 (ζ2)<br>6.92 (ζ3)	7.00
**9 Lys**	8.08	3.87	1.32 (β2)<br>0.96 (β3)	0.27 (γ2)<br>0.10 (γ3)	1.07	2.43 (ε2)<br>2.35 (ε3)	7.23	-
**10 Thr**	7.70	4.18	3.91	0.88	-	-	-	-
**11 Phe**	8.25	4.79	2.67 (β2)<br>2.61 (β3)	-	6.86	7.03	6.97	-
**12 Thr**	8.21	4.24	3.97	0.91	-	-	-	-
**13 Ser**	8.20	4.34	3.68	-	-	-	-	-
**14 Cys**	8.00	4.23	2.98 (β2)<br>2.89 (β3)	-	-	-	-	-

NMR: Data from ^{1}H NMR, TOCSY, NOESY (D_2O, 600 MHz, 285 K).

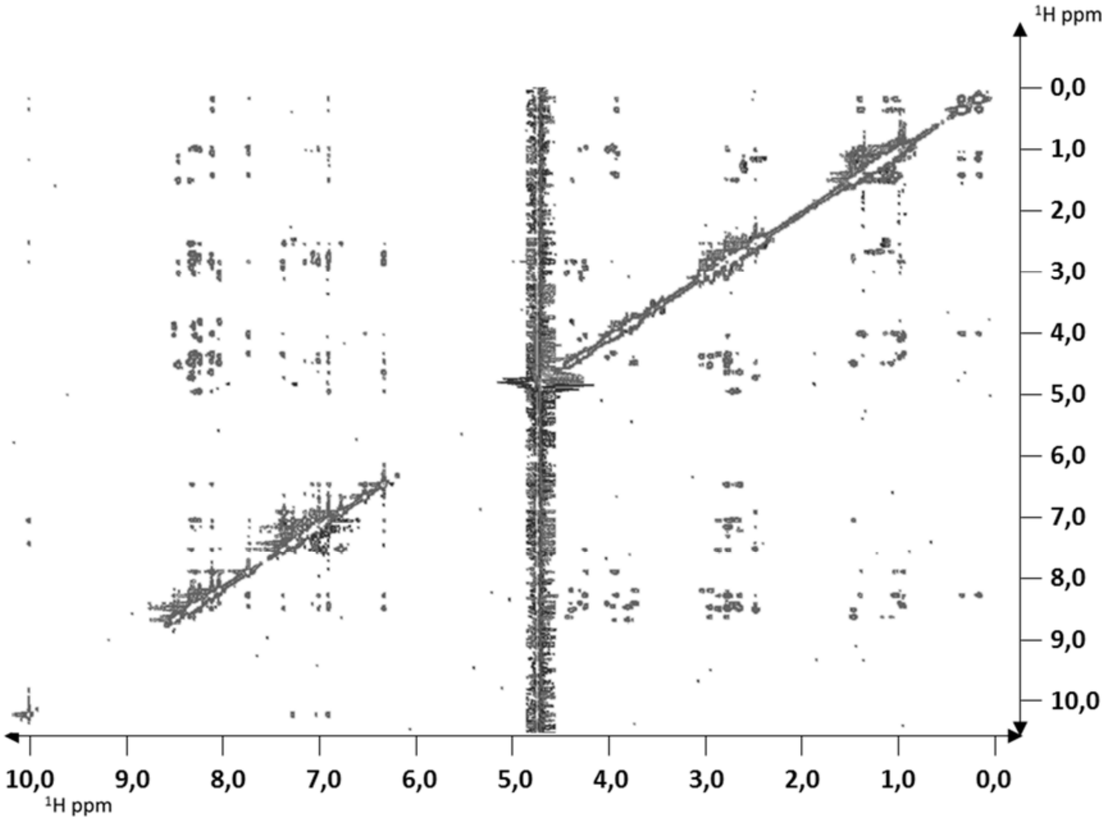

NOESY 350 ms spectra for [L-Dfp6,DTrp8]-SRIF (**1**).

[L-Dfp7,D-Trp8]-SRIF (2): Somatostatin analog **2** was synthesized following the general procedure from 0.20 g of 2-Cl-Trt resin (0.8 mmol/g) and using Fmoc-L-Dfp-OH, affording 0.11 g in 53% yield (99% purity after purification). HPLC: tR = 15.6 [Gradient 25-60%B in 20min, flux: 1 mL.min-1, λ=220 nm]. HRMS: calcd for $C_{76}H_{102}F_2N_{18}O_{19}S_2$: 1672.6978; found, 1672.6964.

	**HN**	**Hα**	**Hβ**	**Hγ**	**Hδ**	**Hε**	**Hζ**	**Hη**
**1 Ala**	-	3.90	1.29	-	-	-	-	-
**2 Gly**	8.47	3.75	-	-	-	-	-	-
**3 Cys**	8.28	4.41	2.89 (β2)<br>2.73 (β3)	-	-	-	-	-
**4 Lys**	8.41	4.27	1.40	1.10 (γ2)<br>1.01 (γ3)	1.26 (δ2)<br>1.20 (δ3)	2.56	7.23	-
**5 Asn**	8.25	4.55	2.42	-	7.31 (δ21)<br>6.71 (δ22)	-	-	-
**6 Phe**	8.16	4.45	2.72 (β2)<br>2.59 (β3)	-	6.71	6.96	6.95	-
**7 Dfp**	8.04	4.34	2.71	-	6.59	-	6.72	-
**8 DTrp**	8.04	4.34	2.89 (β2)<br>2.79 (β3)	-	6.92	10.01 (ε1)<br>7.25 (ε3)	7.36 (ζ2)<br>7.00 (ζ3)	6.91
**9 Lys**	8.11	3.87	1.34 (β2)<br>0.98 (β3)	0.30 (γ2)<br>0.13 (γ3)	1.08	2.44 (ε2)<br>2.37 (ε3)	7.23	-
**10 Thr**	7.68	4.13	3.89	0.87	-	-	-	-
**11 Phe**	8.18	4.66	2.68 (β2)<br>2.59 (β3)	-	6.84	7.03	6.98	-
**12 Thr**	8.11	4.19	3.93	0.92	-	-	-	-
**13 Ser**	8.15	4.31	3.67	-	-	-	-	-
**14 Cys**	8.13	4.36	3.00 (β2)<br>2.88 (β3)	-	-	-	-	-

NMR: Data from ^{1}H NMR, TOCSY, NOESY (D_2O, 600 MHz, 285 K).

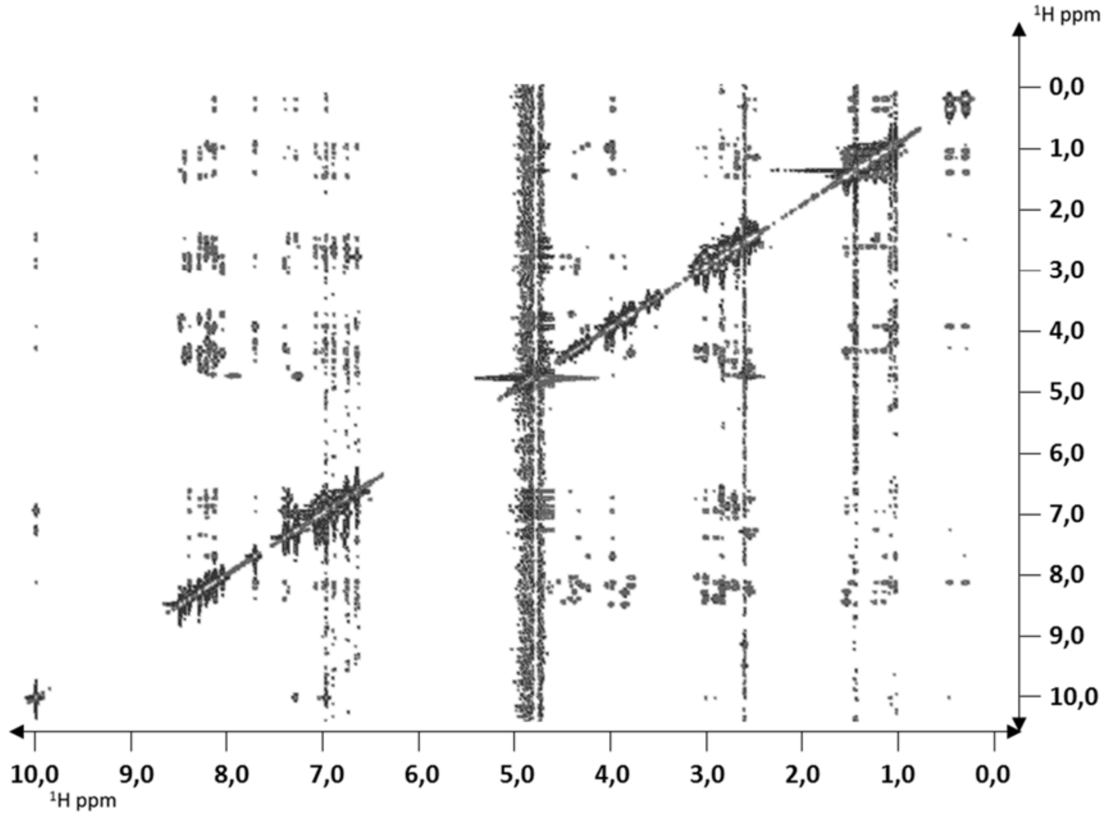

NOESY 350 ms spectra for [L-Dfp7,DTrp8]-SRIF (2).

[D-Trp8,L-Dfp11]-SRIF (3): Somatostatin analog **3** was synthesized following the general procedure from 0.20 g of 2-Cl-Trt resin (0.8 mmol/g) and using Fmoc-L-Dfp-OH, affording 0.14 g in 67% yield (99% purity after purification). HPLC: tR = 17.2 [Gradient 25-60% B in 20 min, flux: 1 mL.min-1, λ=220 nm]. HRMS: calcd. for $C_{76}H_{102}F_2N_{18}O_{19}S_2$: 1672.6978; found, 1672.6894.

	**HN**	**Hα**	**Hβ**	**Hγ**	**Hδ**	**Hε**	**Hζ**	**Hη**
**1 Ala**	7.91	3.89	1.29	-	-	-	-	-
**2 Gly**	8.48	3.75	-	-	-	-	-	-
**3 Cys**	8.28	4.41	2.88 (β2)<br>2.76 (β3)	-	-	-	-	-
**4 Lys**	8.40	4.26	1.43	1.13 (γ2)<br>1.03 (γ3)	1.30	2.63	7.27	-
**5 Asn**	8.17	4.51	2.43	-	7.33 (δ21)<br>6.72 (δ22)	-	-	-
**6 Phe**	8.04	4.51	2.71 (β2)<br>2.56 (β3)	-	6.76	6.98	6.95	-
**7 Phe**	8.05	4.34	2.72	-	6.98	7.12	7.07	-
**8 DTrp**	8.28	4.20	2.83	-	6.88	9.98 (ε1)<br>7.24 (ε3)	7.24 (ζ2)<br>6.91 (ζ3)	7.00
**9 Lys**	8.05	3.87	1.33 (β2)<br>0.99 (β3)	0.30 (γ2)<br>0.14 (γ3)	1.08	2.44 (ε2)<br>2.39 (ε3)	7.23	-
**10 Thr**	7.79	4.10	3.92	0.89	-	-	-	-
**11 Dfp**	8.35	4.59	2.72 (β2)<br>2.61 (β3)	-	6.41	-	6.53	-
**12 Thr**	8.11	4.17	3.91	0.90	-	-	-	-
**13 Ser**	8.15	4.26	3.65	-	-	-	-	-
**14 Cys**	8.11	4.31	2.98 (β2)<br>2.85 (β3)	-	-	-	-	-

NMR: Data from ^{1}H NMR, TOCSY, NOESY (D_2O, 600 MHz, 285 K).

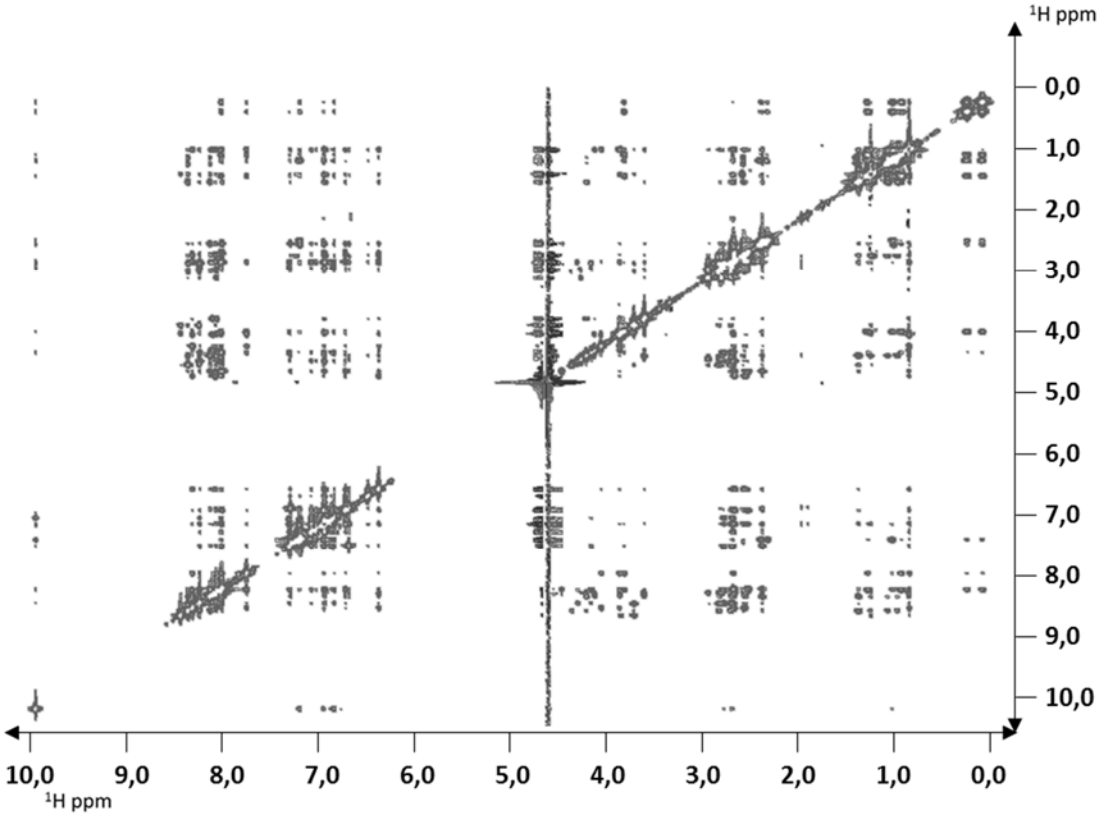

NOESY 350 ms spectra for [DTrp8,L-Dfp11]-SRIF (**3**).

[L-Dfp6,L-Msa7,D-Trp8]-SRIF (4): Somatostatin analog **4** was synthesized following the general procedure from 1.00 g of 2-Cl-Trt resin (1.60 mmol/g), and using using Fmoc-L-Dfp-OH and Fmoc-L-Msa-OH, affording 0.56 mg of pure peptide. LC-MS: t_R = 6.97 [gradient 5-100%B in 8 min, flux: 0.3 ml.min^{-1}, λ=210-400 nm]. HRMS: calcl. for $C_{79}H_{108}F_2N_{18}O_{19}S_2$: 1714.7448; found, 1714.7439.

	HN	Hα	Hβ	Hγ	Hδ	Hε	Hζ	Hη
**1 Ala**	7.91	3.90	1.30	-	-	-	-	-
**2 Gly**	8.47	3.75	-	-	-	-	-	-
**3 Cys**	8.27	4.37	2.91 (β2)<br>2.71 (β3)	-	-	-	-	-
**4 Lys**	8.42	4.47	1.36 (β2)<br>1.30 (β3)	1.07 (γ2)<br>0.97 (γ3)	1.64	2.48	7.15	-
**5 Asn**	8.39	4.61	2.46 (β2)<br>2.29 (β3)	-	7.34 (δ21)<br>6.76 (δ22)	-	-	-
**6 Dfp**	8.28	4.60	2.72 (β2)<br>2.58 (β3)	-	6.35	-	6.45	-
**7 Msa**	8.21	4.37	2.81 (β2)<br>2.76 (β3)	-	2.03 (Hφ)	6.66	-	1.93
**8 DTrp**	8.32	4.29	2.85	-	6.89	10.01 (ε1)<br>7.38 (ε3)	7.26 (ζ2)<br>6.94 (ζ3)	7.02
**9 Lys**	8.15	4.46	1.40 (β2)<br>0.99 (β3)	0.27 (γ2)<br>0.05 (γ3)	1.08	2.45 (ε2)<br>2.37 (ε3)	7.24	-
**10 Thr**	7.85	4.46	3.97	0.91	-	-	-	-
**11 Phe**	8.17	5.07	2.48	-	6.82	7.05	6.98	-
**12 Thr**	8.36	4.31	4.04	0.96	-	-	-	-
**13 Ser**	8.35	4.35	3.75 (β2)<br>3.70 (β3)	-	-	-	-	-
**14 Cys**	8.07	4.34	3.02 (β2)	-	-	-	-	-

2.94 (β3)

NMR: Data from ^{1}H NMR, TOCSY, NOESY (D_2O, 600 MHz, 285 K).

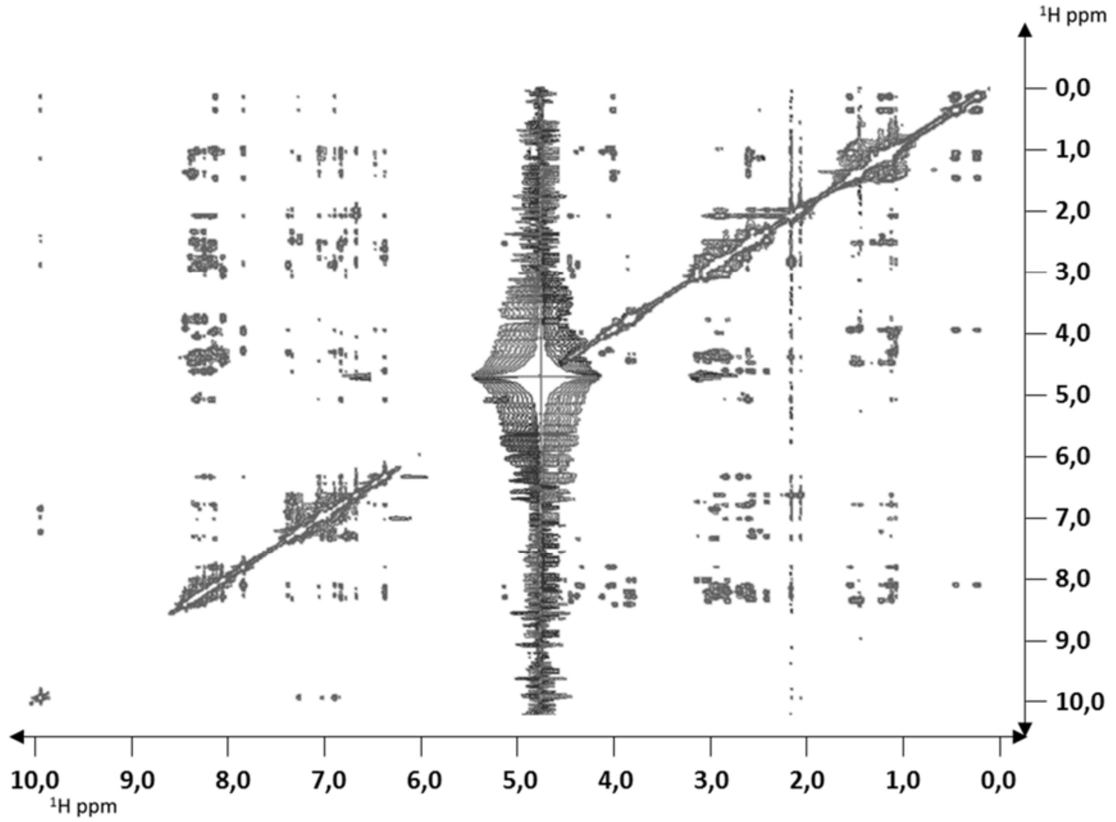

NOESY 350 ms spectra for [L-Dfp6,L-Msa7,DTrp8]-SRIF (**4**).

[L-Msa7,D-Trp8,L-Dfp11]-SRIF (5): Somatostatin analog **5** was synthesized following the general procedure from 1.00 g of 2-Cl-Trt resin (1.60 mmol/g), and using using Fmoc-L-Dfp-OH and Fmoc-L-Msa-OH, affording 12,6 mg of pure peptide. LC-MS: t_R = 7.28 [gradient 5-100%B in 8 min, flux: 0.3 $ml.min^{-1}$, λ=210-400 nm]. HRMS: calcl. for $C_{79}H_{108}F_2N_{18}O_{19}S_2$: 1714.7448; found 1714.7421.

	HN	Hα	Hβ	Hγ	Hδ	Hε	Hζ	Hη
**1 Ala**	7.91	3.90	1.29	-	-	-	-	-
**2 Gly**	8.51	3.75	-	-	-	-	-	-
**3 Cys**	8.27	4.38	2.92 (β2)<br>2.71 (β3)	-	-	-	-	-
**4 Lys**	8.39	4.40	1.39 (β2)<br>1.28 (β3)	1.10 (γ2)<br>0.97 (γ3)	1.33	2.56	-	-
**5 Asn**	8.28	4.58	2.44 (β2)<br>2.31 (β3)	-	7.35 (δ21)<br>6.75 (δ22)	-	-	-
**6 Phe**	8.15	4.56	2.69 (β2)<br>2.57 (β3)	-	6.77	6.99	6.88	-
**7 Msa**	8.19	4.38	2.79	-	2.03 (Hφ)	6.66	-	1.93
**8 DTrp**	8.24	4.28	2.84	-	6.87	10.01 (ε1)<br>7.36 (ε3)	7.26 (ζ2)<br>6.92 (ζ3)	7.00
**9 Lys**	8.11	3.91	1.38 (β2)<br>1.00 (β3)	0.30 (γ2)<br>0.10 (γ3)	1.08	2.45 (ε2)<br>2.38 (ε3)	-	-
**10 Thr**	7.87	4.19	3.97	0.92	-	-	-	-
**11 Dfp**	8.27	4.90	2.48 (β2)<br>2.43 (β3)	-	6.36	-	6.54	-
**12 Thr**	8.29	4.23	3.99	0.94	-	-	-	-
**13 Ser**	8.26	4.38	3.69	-	-	-	-	-
**14 Cys**	7.96	4.22	2.97	-	-	-	-	-

NMR: Data from ^1H NMR, TOCSY, NOESY (D_2O, 600 MHz, 285 K).

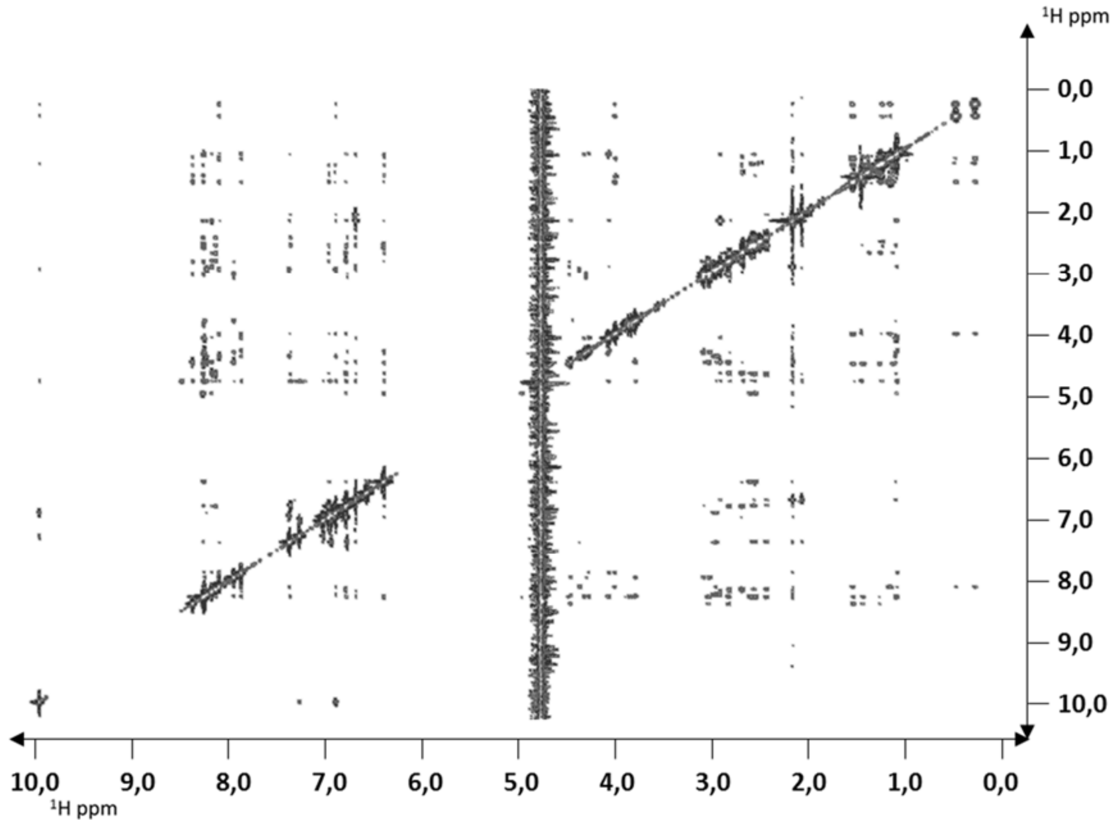

NOESY 350 ms spectra for [L-Msa7,DTrp8,L-Dfp11]-SRIF (**5**).

[L-Dfp6,11,L-Msa7,D-Trp8]-SRIF (6): Somatostatin analog **6** was synthesized following the general procedure from 0.05 g of 2-Cl-Trt resin (0.8 mmol/g) and using Fmoc-L-Dfp-OH and Fmoc-L-Msa-OH, affording 0.05 g in 61% yield (99% purity after purification). HPLC: tR = 16.1 [Gradient 25-60% B in 20 min, flux: 1 mL.min^{-1}, λ=220 nm]. HRMS: calcd. for $C_{79}H_{106}F_4N_{18}O_{19}S_2$: 1756.7917; found, 1756.7923.

	HN	Hα	Hβ	Hγ	Hδ	Hε	Hζ	Hη
**1 Ala**	7.90	3.89	1.30	-	-	-	-	-
**2 Gly**	8.48	3.75	-	-	-	-	-	-
**3 Cys**	8.27	4.37	2.90 (β2)<br>2.73 (β3)	-	-	-	-	-
**4 Lys**	8.39	4.43	1.39	1.12 (γ2)<br>1.02 (γ3)	1.23	2.59	7.25	-
**5 Asn**	8.27	4.58	2.45 (β2)<br>2.30 (β3)	-	7.34 (δ21)<br>6.76 (δ22)	-	-	-
**6 Dfp**	8.16	4.64	2.70 (β2)<br>2.55 (β3)	-	6.36	-	6.47	-
**7 Msa**	8.21	4.35	2.80	-	2.03 (Hφ)	6.66	-	1.93
**8 DTrp**	8.28	4.28	2.82	-	6.85	10.00 (ε1)<br>7.36 (ε3)	7.36 (ζ2)<br>7.00 (ζ3)	6.93
**9 Lys**	8.10	3.90	1.38 (β2)<br>0.97 (β3)	0.27 (γ2)<br>0.06 (γ3)	1.07	2,45 (ε2)<br>2,36 (ε3)	7,23	-
**10 Thr**	7.93	4.20	3.99	0.92	-	-	-	-
**11 Dfp**	8.28	4.97	2.55	-	6.40	-	6.50	-
**12 Thr**	8.28	4.28	4.00	0.93	-	-	-	-
**13 Ser**	8.29	4.38	3.69	-	-	-	-	-
**14 Cys**	8.01	4.24	2.98 (β2)<br>2.90 (β3)	-	-	-	-	-

NMR: Data from ^{1}H NMR, TOCSY, NOESY (D_2O, 600 MHz, 285 K).

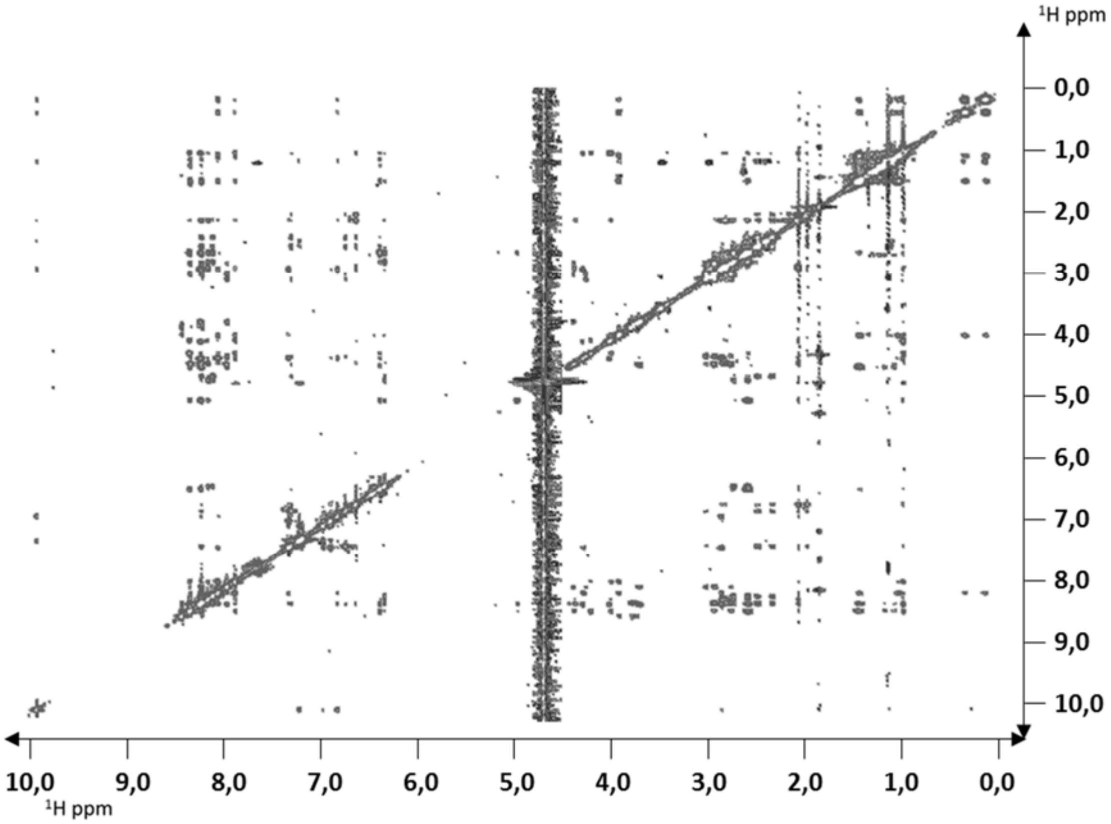

NOESY 350 ms spectra for [L-Dfp6,11,L-Msa7,DTrp8]-SST (**6**).

NMR and Computational methods: All NMR data was processed with NMRPipe.[3] Cara[4] was used to assign the spectra. Distance restraints derived from fully assigned peaks in NOESY experiments were used for structure calculation. The structures were calculated with the programs CNS[5] and StructCalc.[6] Statistics from the analysis are shown the tables. PyMOL[7] was used to visualize the structures and generate the figures.

Structural Statistics for the 20 Lowest Energy Structures of [L-Dfp6,D-Trp8]-SRIF (**1**).

Distance Restricions:

Intraresidual : 0
Sequential: 48
Medium-range (1<dist<=4) : 27
Long-range (dist>4) : 28
Total: 99
Dihedral angle restrictions : 22

Statistics for 20 best structures

**Energies (kcal.mol^{-1}):**	**RMSD[a] :**
Total energy : -357.2 +/- 8.1	Bonds (Å) : 0.005913 +/- 0.0003035
Van der Waals : -38.62 +/- 4.65	Angles (º): 0.6805 +/- 0.02805
Electrostatic : -447.1 +/- 10.2	Impropers (º): 1.398 +/- 0.1449
Bonds : 7.991 +/- 0.822	Dihedrals (º): 41.25 +/- 0.5487
Angles: 28.54 +/- 2.35	NOEs : 0.01067 +/- 0.0007425

[a]R.M.S. Deviation between the ensemble of the 20 structures with the lowest energy and the lowest energy structure

[3] F. Delaglio, S. Grzesiek, G. Vuister, G. Zhu, J. Pfeifer, A. Bax *J. Biomol. NMR* **1995**, *6*, 277-293.
[4] R. Keller, The Computer Aided Resonance Assignment Tutorial, 1st ed.CANTINA.Verlag, 2004.
[5] A. T. Brunger, P. D. Adams, G. M. Clore, W. L. DeLano, P. Gros, R. W. Grosse-Kunstleve, J. S. Jiang, J. Kuszewski, M. Nilges, N. S. Pannu, R. J. Read, L. M. Rice, T. Simonson, G. L. Warren *Acta Crystallogr. D Biol. Crystallogr.* **1998**, *54*, 905-921.
[6] P. Martín-Malpartida, M. J. Macías, unpublished data.
[7] W. L. DeLano. The Pymol molecular graphics system. Palo Alto, CA: DeLano Scientific, **2002**. USA.

Structural Statistics for the 20 lowest energy structures of [L-Dfp7,D-Trp8]-SRIF (**2**).

Distance Restricions:

Intraresidual : 0

Sequential: 63

Medium-range (1<dist<=4) : 22

Long-range (dist>4) : 10

Total: 95

Dihedral angle restrictions : 19

Statistics for 20 best structures

**Energies (kcal.mol^{-1}):**	**RMSD[a] :**
Total energy : -195.3 +/- 14.12	Bonds (Å) : 0.01357 +/- 0.0008174
Van der Waals : -13.57 +/- 7.295	Angles (º): 1.176 +/- 0.03831
Electrostatic : -441.3 +/- 15.76	Impropers (º): 1.91 +/- 0.1157.
Bonds : 42.16 +/- 5.115	Dihedrals (º): 42.59 +/- 0.4219
Angles : 85.18 +/- 5.597	NOEs : 0.02691 +/- 0.002473

Structural Statistics for the 20 lowest energy structures of [D-Trp8,L-Dfp11]-SRIF (**3**).

Distance Restricions:

Intraresidual : 0

Sequential: 72

Medium-range (1<dist<=4) : 13

Long-range (dist>4) : 21

Total: 106

Dihedral angle restrictions : 10

Statistics for 20 best structures

**Energies (kcal.mol^{-1}):**	**RMSD[a] :**
Total energy : 4.57 +/- 17.83	Bonds (Å) : 0.01357 +/- 0.0008174
Van der Waals : 5.861 +/- 8.52	Angles (º): 1.176 +/- 0.03831
Electrostatic : -407.4 +/- 11.8	Impropers (º): 3.478 +/- 0.4543
Bonds : 45.91 +/- 6.96	Dihedrals (º): 42.51 +/- 0.4808
Angles : 182.4 +/- 14.9	NOEs : 0.02691 +/- 0.002473

Structural Statistics for the 20 lowest energy structures of [L-Dfp6,L-Msa7,D-Trp8]-SRIF (**4**).

Distance Restricions:

Intraresidual : 0

Sequential: 48

Medium-range (1<dist<=4) : 17

Long-range (dist>4) : 21

Total: 86

Dihedral angle restrictions : 22

Statistics for 20 best structures

**Energies (kcal.mol^{-1}):**	**RMSD[a] :**
Total energy : -135.4 +/- 7.376	Bonds (Å) : 0.01037 +/- 0.0004011
Van der Waals : -19.74 +/- 4.517	Angles (º): 1.467 +/- 0.02504
Electrostatic : -438.4 +/- 14.12	Impropers (º): 3.247 +/- 0.2407
Bonds : 25.53 +/- 1.983	Dihedrals (º): 41.78 +/- 0.3209
Angles : 138.3 +/- 4.741	NOEs : 0.0214 +/- 0.0008323

Structural Statistics for the 20 lowest energy structures of [L-Msa7,D-Trp8,L-Dfp11]-SRIF (**5**).

Distance Restricions:

Intraresidual : 0

Sequential: 96

Medium-range (1<dist<=4) : 63

Long-range (dist>4) : 64

Total: 223

Dihedral angle restrictions : 22

Statistics for 20 best structures

**Energies (kcal.mol^{-1}):**	**RMSD[a] :**
Total energy : -16.26 +/- 9.679	Bonds (Å) : 0.01213 +/- 0.0003598
Van der Waals : 11.33 +/- 4.447	Angles (º): 1.534 +/- 0.02475
Electrostatic : -397.5 +/- 7.754	Impropers (º): 3.953 +/- 0.1101
Bonds : 34.61 +/- 2.051	Dihedrals (º): 42.61 +/- 0.3222
Angles : 150.2 +/- 4.87	NOEs : 0.01277 +/- 0.0004352

Structural Statistics for the 20 lowest energy structures of [L-Dfp6,11,L-Msa7,D-Trp8]-SRIF (**6**).

Distance Restricions:

Intraresidual : 0

Sequential: 53

Medium-range (1<dist<=4) : 28

Long-range (dist>4) : 11

Total: 92

Dihedral angle restrictions : 22

Statistics for 20 best structures

**Energies (kcal.mol^{-1}):**	**RMSD[a] :**
Total energy : -308.4 +/- 9.2	Bonds (Å) : 0.005061 +/- 0.0003145
Van der Waals : -308.4 +/- 9.2	Angles (º): 1.104 +/- 0.01969
Electrostatic : -452.8 +/- 9.6	Impropers (º):1.749 +/- 0.2341
Bonds : 6.093 +/- 0.758	Dihedrals (º): 40.13 +/- 0.3457
Angles: 78.41 +/- 2.817	NOEs : 0.005899 +/- 0.001354

Binding assays: All peptides were subjected to anion exchange (F_3CCOO^- → AcO^-) by using DOWEX resin (Dowex Monosphere 550A (OH)). Biological tests were carried out with previously purified peptides containing acetate counter-ion.

Receptor Ligand-Binding Assay: All receptor-binding assays were performed with membranes isolated from CHO-K1 cells expressing the cloned human SRIF-14 receptor, developed by the company Eurofins Panlab S.A. The assay buffer consisted of 50 mM Tris (pH 7.5) with 1 nM EGTA, 5 mM MgCl2, leupeptin (10 µg/ml), pepstatin (10 µg/ml), bacitracin (200 µg/ml), aprotinin (0.5 µg/ml) and 0.2% BSA. CHO-K1 cell membranes, radiolabeled SRIF-14 and unlabeled test compounds were diluted in this assay buffer. All assays were performed in 96-well polypropylene plates. Ten micrograms of membrane proteins were incubated with 0.1nM of 125I-Tyr11-SRIF (specific activity- 2000 Ci/mmol) in the presence or absence of various concentrations of unlabeled peptides (1pM-1000 nM) in a total volume of 200 µl, for 1 h at 30ºC. The binding reaction was terminated by vacuum filtration over Whatman GF/F glass fibre filters previously pre-soaked in 0.5% (w/v) polyethyleneimine and 0.2% bovine serum albumin, using a 98-well harvester (Inotech). The filters were washed with ice-cold 50 mM Tris-HCl (pH 7.5) and dried, after which scintillator sheets were melted onto the filter and the bound radioactivity was analyzed in a liquid scintillation counter (microβ plus, Wallac). Specific binding was defined as the total ^{125}I-Tyr11-SRIF binding minus the amount bound in the presence of 1000 nM SRIF (non-specific binding).
